# Detecting overlapping coding sequences in virus genomes

**DOI:** 10.1186/1471-2105-7-75

**Published:** 2006-02-16

**Authors:** Andrew E Firth, Chris M Brown

**Affiliations:** 1 Department of Biochemistry, University of Otago, PO Box 56, Dunedin, New Zealand

## Abstract

**Background:**

Detecting new coding sequences (CDSs) in viral genomes can be difficult for several reasons. The typically compact genomes often contain a number of overlapping coding and non-coding functional elements, which can result in unusual patterns of codon usage; conservation between related sequences can be difficult to interpret – especially within overlapping genes; and viruses often employ non-canonical translational mechanisms – e.g. frameshifting, stop codon read-through, leaky-scanning and internal ribosome entry sites – which can conceal potentially coding open reading frames (ORFs).

**Results:**

In a previous paper we introduced a new statistic – MLOGD (Maximum Likelihood Overlapping Gene Detector) – for detecting and analysing overlapping CDSs. Here we present (a) an improved MLOGD statistic, (b) a greatly extended suite of software using MLOGD, (c) a database of results for 640 virus sequence alignments, and (d) a web-interface to the software and database. Tests show that, from an alignment with just 20 mutations, MLOGD can discriminate non-overlapping CDSs from non-coding ORFs with a typical accuracy of up to 98%, and can detect CDSs overlapping known CDSs with a typical accuracy of 90%. In addition, the software produces a variety of statistics and graphics, useful for analysing an input multiple sequence alignment.

**Conclusion:**

MLOGD is an easy-to-use tool for virus genome annotation, detecting new CDSs – in particular overlapping or short CDSs – and for analysing overlapping CDSs following frameshift sites. The software, web-server, database and supplementary material are available at .

## Background

Methods for finding protein-coding sequences (CDSs) in prokaryotes and eukaryotes are well-developed. Algorithms generally make use of combinations of the following signatures of CDSs: (a) codon or dicodon bias etc., (b) conservation between species, (c) similarity to known sequences, (d) presence of open reading frames (ORFs), splice sites etc., and (e) expression in cDNA/EST libraries [1].

In virus genomes, however, the situation can be complicated by a number of factors, that may lead to decreased sensitivity: (a) virus genomes are often too small (e.g. < 10 kb) to obtain codon usage statistics and, in any case, the compact genomes often contain overlapping coding and non-coding functional elements that can result in unusual codon usage patterns; (b) regions of high conservation between related sequences may not necessarily be coding and, where CDSs and/or non-coding functional elements overlap, conservation may only reveal the presence of one of the overlapping pair; (c) new virus types often contain novel CDSs, dissimilar to previously annotated CDS; and (d) viruses can employ a variety of non-canonical translational mechanisms – e.g. frameshifting, stop codon read-through, leaky-scanning and internal ribosome entry sites.

Comparative genomics is a particularly useful way to detect new CDSs in virus genomes, because many sequenced virus genomes, covering a useful range of diversity (i.e. sequence divergence), are available. In its simplest form, a comparative genomics approach consists of looking for genome regions that are more conserved than average between related sequences. Such an approach may fail to distinguish CDSs from other conserved elements. A more advanced approach is to look for the particular mutation patterns associated with CDSs – e.g. the CRITICA software [2], or pair hidden Markov models [3]. However, previous such algorithms have not dealt adequately with the case of overlapping CDSs.

In a previous paper [4] we introduced a probabilistic model for the mutation patterns associated with non-coding, single-coding and double-coding regions of a multiple sequence alignment, and a maximum likelihood statistic – called MLOGD – for predicting whether a new query ORF is coding or non-coding. Here we present (a) an improved MLOGD statistic, (b) a greatly extended suite of software using MLOGD (70% is new relative to [4], the rest has been substantially revised), (c) a database of results in virus genomes, and (d) a web-interface to the software and database.

## Implementation

### The MLOGD statistic

Given an input sequence alignment, a null model of the CDS annotation (i.e. the known CDSs in some chosen reference sequence) and an alternate model (i.e. the known CDSs plus a new putative CDS), the MLOGD statistic is an estimate of the relative probabilities of obtaining the observed pattern of mutations across the alignment under each of the null and alternate models. In this subsection we first describe how the MLOGD statistic is calculated for a pairwise sequence alignment. Then we describe how this is extended to a multiple sequence alignment. More extensive notes are given on the website.

#### MLOGD statistic for a two-sequence alignment

Given two aligned sequences *S*_1 _and *S*_2_, we estimate the probability that *S*_1 _mutates to *S*_2_, after time *t*, by

log⁡P(S1→S2;t,M)=∑Nnucleotidesk=1log⁡P(N1k→N2k;t,mk),     (1)
 MathType@MTEF@5@5@+=feaafiart1ev1aaatCvAUfKttLearuWrP9MDH5MBPbIqV92AaeXatLxBI9gBaebbnrfifHhDYfgasaacH8akY=wiFfYdH8Gipec8Eeeu0xXdbba9frFj0=OqFfea0dXdd9vqai=hGuQ8kuc9pgc9s8qqaq=dirpe0xb9q8qiLsFr0=vr0=vr0dc8meaabaqaciaacaGaaeqabaqabeGadaaakqGabeqaaGRabaGagiiBaWMaei4Ba8Maei4zaCMaemiuaaLaeiikaGIaem4uam1aaSbaaSqaaiabigdaXaqabaGccqGHsgIRcqWGtbWudaWgaaWcbaGaeGOmaidabeaakiabcUda7iabdsha0jabcYcaSiabd2eanjabcMcaPiabg2da9aqaaiaaxMaadaaeabqaauaabaqaceaaaeaacqWGobGtdaWgaaWcbaGaeeOBa4MaeeyDauNaee4yamMaeeiBaWMaeeyzauMaee4Ba8MaeeiDaqNaeeyAaKMaeeizaqMaeeyzauMaee4CamhabeaaaOqaaiabdUgaRjabg2da9iabigdaXaaaaSqabeqaniabggHiLdGccyGGSbaBcqGGVbWBcqGGNbWzcqWGqbaucqGGOaakcqWGobGtdaqhaaWcbaGaeGymaedabaGaem4AaSgaaOGaeyOKH4QaemOta40aa0baaSqaaiabikdaYaqaaiabdUgaRbaakiabcUda7iabdsha0jabcYcaSiabd2gaTnaaCaaaleqabaGaem4AaSgaaOGaeiykaKIaeiilaWIaaCzcaiaaxMaadaqadaqaaiabigdaXaGaayjkaiaawMcaaaaaaa@7224@

where N1k
 MathType@MTEF@5@5@+=feaafiart1ev1aaatCvAUfKttLearuWrP9MDH5MBPbIqV92AaeXatLxBI9gBaebbnrfifHhDYfgasaacH8akY=wiFfYdH8Gipec8Eeeu0xXdbba9frFj0=OqFfea0dXdd9vqai=hGuQ8kuc9pgc9s8qqaq=dirpe0xb9q8qiLsFr0=vr0=vr0dc8meaabaqaciaacaGaaeqabaqabeGadaaakeaacqWGobGtdaqhaaWcbaGaeGymaedabaGaem4AaSgaaaaa@304D@ and N2k
 MathType@MTEF@5@5@+=feaafiart1ev1aaatCvAUfKttLearuWrP9MDH5MBPbIqV92AaeXatLxBI9gBaebbnrfifHhDYfgasaacH8akY=wiFfYdH8Gipec8Eeeu0xXdbba9frFj0=OqFfea0dXdd9vqai=hGuQ8kuc9pgc9s8qqaq=dirpe0xb9q8qiLsFr0=vr0=vr0dc8meaabaqaciaacaGaaeqabaqabeGadaaakeaacqWGobGtdaqhaaWcbaGaeGOmaidabaGaem4AaSgaaaaa@304F@ are the nucleotides in *S*_1 _and *S*_2 _at the *k*th alignment position (see website for treatment of alignment gaps); *P*(N1k
 MathType@MTEF@5@5@+=feaafiart1ev1aaatCvAUfKttLearuWrP9MDH5MBPbIqV92AaeXatLxBI9gBaebbnrfifHhDYfgasaacH8akY=wiFfYdH8Gipec8Eeeu0xXdbba9frFj0=OqFfea0dXdd9vqai=hGuQ8kuc9pgc9s8qqaq=dirpe0xb9q8qiLsFr0=vr0=vr0dc8meaabaqaciaacaGaaeqabaqabeGadaaakeaacqWGobGtdaqhaaWcbaGaeGymaedabaGaem4AaSgaaaaa@304D@ → N2k
 MathType@MTEF@5@5@+=feaafiart1ev1aaatCvAUfKttLearuWrP9MDH5MBPbIqV92AaeXatLxBI9gBaebbnrfifHhDYfgasaacH8akY=wiFfYdH8Gipec8Eeeu0xXdbba9frFj0=OqFfea0dXdd9vqai=hGuQ8kuc9pgc9s8qqaq=dirpe0xb9q8qiLsFr0=vr0=vr0dc8meaabaqaciaacaGaaeqabaqabeGadaaakeaacqWGobGtdaqhaaWcbaGaeGOmaidabaGaem4AaSgaaaaa@304F@; *t*, *m*^*k*^) is calculated using nucleotide, codon and amino acid substitution matrices, as described in [4] (with the obvious extension for the non-coding model); *M *is the null or alternate model; and *m*^*k *^is the coding status (non-coding, single-coding or double-coding) at the *k*th alignment position, according to the relevant model *M *(defined on the chosen reference sequence).

We define the pairwise sequence divergence, Λ, to be the total number of point nucleotide differences between *S*_1 _and *S*_2_, and we determine *t *numerically for each of the null and alternate models by requiring the expected number of mutations between *S*_1 _and *S*_2_, under the model, to equal the observed number of mutations, Λ.

The log likelihood ratio of the two models is

log⁡(LR)=log⁡P(S1→S2;t=talt,M=alt)−log⁡P(S1→S2;t=tnull,M=null).     (2)
 MathType@MTEF@5@5@+=feaafiart1ev1aaatCvAUfKttLearuWrP9MDH5MBPbIqV92AaeXatLxBI9gBaebbnrfifHhDYfgasaacH8akY=wiFfYdH8Gipec8Eeeu0xXdbba9frFj0=OqFfea0dXdd9vqai=hGuQ8kuc9pgc9s8qqaq=dirpe0xb9q8qiLsFr0=vr0=vr0dc8meaabaqaciaacaGaaeqabaqabeGadaaakeaafaqaaeGadaaabaGagiiBaWMaei4Ba8Maei4zaCMaeiikaGIaeeitaWKaeeOuaiLaeeykaKcabaGaeyypa0dabaGagiiBaWMaei4Ba8Maei4zaCMaemiuaaLaeiikaGIaem4uam1aaSbaaSqaaiabigdaXaqabaGccqGHsgIRcqWGtbWudaWgaaWcbaGaeGOmaidabeaakiabcUda7iabdsha0jabg2da9iabdsha0naaCaaaleqabaGaeeyyaeMaeeiBaWMaeeiDaqhaaOGaeiilaWIaemyta0Kaeyypa0JaeeyyaeMaeeiBaWMaeeiDaqNaeeykaKIaeyOeI0cabaaabaaabaGagiiBaWMaei4Ba8Maei4zaCMaemiuaaLaeiikaGIaem4uam1aaSbaaSqaaiabigdaXaqabaGccqGHsgIRcqWGtbWudaWgaaWcbaGaeGOmaidabeaakiabcUda7iabdsha0jabg2da9iabdsha0naaCaaaleqabaGaeeOBa4MaeeyDauNaeeiBaWMaeeiBaWgaaOGaeiilaWIaemyta0Kaeyypa0JaeeOBa4MaeeyDauNaeeiBaWMaeeiBaWMaeeykaKIaeeOla4caaiaaxMaacaWLjaWaaeWaaeaacqaIYaGmaiaawIcacaGLPaaaaaa@7A2C@

If log(LR) is positive, then the observed mutations between *S*_1 _and *S*_2 _are more consistent with the alternate model. If log(LR) is negative, then the observed mutations are more consistent with the null model.

#### MLOGD statistic for a multiple sequence alignment

The MLOGD statistic, ∑_tree _log(LR), is calculated for a multiple sequence alignment using the following procedure. First a phylogenetic tree is constructed using standard software (e.g. PHYLIP, [5]). The (unrooted) tree is used to select a list of sequence pairs tracing round the outside of the tree (figure on website). Such a set of pairwise comparisons covers each branch of the tree precisely twice. The MLOGD statistic, log(LR), is calculated for each of the sequence pairs in the list, summed up over all the pairs, and divided by two, to give the MLOGD statistic for the multiple sequence alignment, ∑_tree _log (LR). Similarly, the total number of mutations, ∑_tree _Λ across the phylogenetic tree is the sum of Λ values for each sequence pair, divided by two.

### Input data

The required input data for MLOGD are a multiple sequence alignment of related sequences, a list of known CDSs (possibly none) in a chosen reference sequence, and a phylogenetic tree. Circular genomes are fully supported. For viruses, useful sets of related sequences may be obtained from the NCBI Viral Genomes Project website [6]. There are tools on the web-server to help produce a suitable alignment and phylogenetic tree.

### Operation modes

The MLOGD software has three operation modes, described below. The 'Test input query CDSs' option can be used to test a specific query CDS (e.g. an ORF that has not previously been annotated as a CDS). The 'Find and test all non-annotated ORFs' and 'Six-frame sliding window plots' options can be used to search a whole input alignment for new CDSs.

#### Test input query CDSs

Here MLOGD calculates the null versus alternate model likelihood ratio statistics, where the null model is that the query ORF is non-coding, while the alternate model is that the query ORF is coding (both the null and alternate models include all the annotated CDSs).

**Figure 1:**
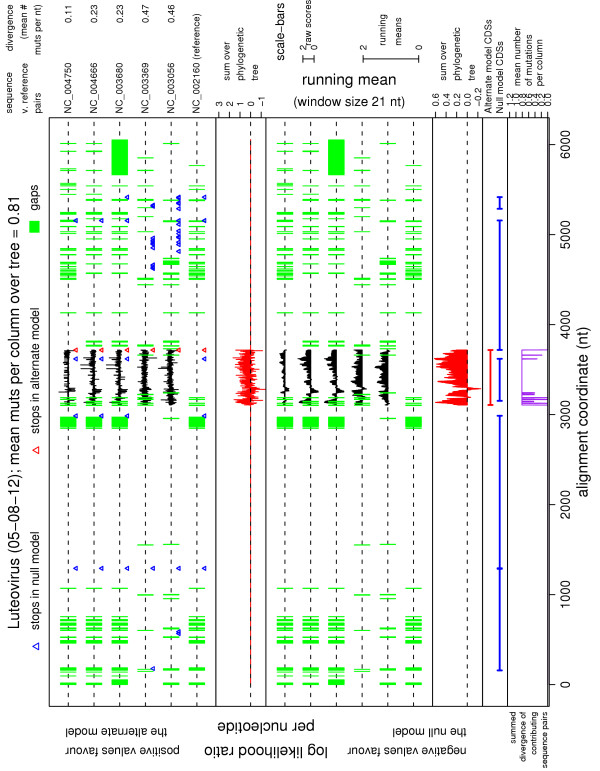
**Nucleotide-by-nucleotide plot**. Example output nucleotide-by-nucleotide plot for the 'Test input query CDSs' option. Luteovirus, six sequences [GenBank:NC_002160, GenBank:NC_003056, GenBank:NC_003369, GenBank:NC_003680, GenBank:NC_004666, GenBank:NC_004750], with NC_002160 as the reference sequence. NC_002160 has six annotated CDSs. CDS3 was used as the query CDS and the remaining five CDSs were taken as the known CDSs. The first panel displays the raw log(LR) statistics at each alignment position. There is a separate track for each reference – non-reference sequence pair (labelled at the right, together with the pairwise divergences). Gaps, and stop codons in each of the null and alternate model CDSs, for each sequence, are marked on the appropriate tracks. The second panel displays the ∑_tree _log (LR) statistic at each alignment position. The third and fourth panels display sliding window means of the statistics in the first and second panels, respectively. The fifth panel shows the locations of the null and alternate model CDSs. The sixth panel shows the summed mean sequence divergence (mutations per nt) for the sequence pairs that contribute to the ∑_tree _log (LR) statistic at each alignment position. This is a measure of the information available at each alignment position (e.g. partially gapped regions have lower summed mean sequence divergence). (See website for more details.) The predominantly positive values in the fourth panel show that CDS3 is functionally constrained over the majority of its length.

The output includes:

1. A table of log(LR) statistics for each reference – non-reference sequence pair.

2. A plot of the log(LR) statistic for each reference – non-reference sequence pair and summed over the phylogenetic tree. On the web-server there is a link to generate Monte Carlo simulated sequences under the same null and alternate models. The simulations are used to estimate confidence limits on the log(LR) statistics. (Figures not shown.)

3. A nucleotide-by-nucleotide plot of the log(LR) statistic for each reference – non-reference sequence pair, the sum over the phylogenetic tree, and running means of the same (Figure 1). On the web-server there are links to zoom in on the plot and/or adjust the running-mean window size.

#### Find and test all non-annotated ORFs

The 'Find and test all non-annotated ORFs' option finds all non-annotated ORFs longer than a specified minimum length, and produces the same statistics and plots as the 'Test input query CDSs' option for each of these ORFs. The user may select 'start-stop' ORFs or 'stop-stop' ORFs.

#### Six-frame sliding window plots

**Figure 2:**
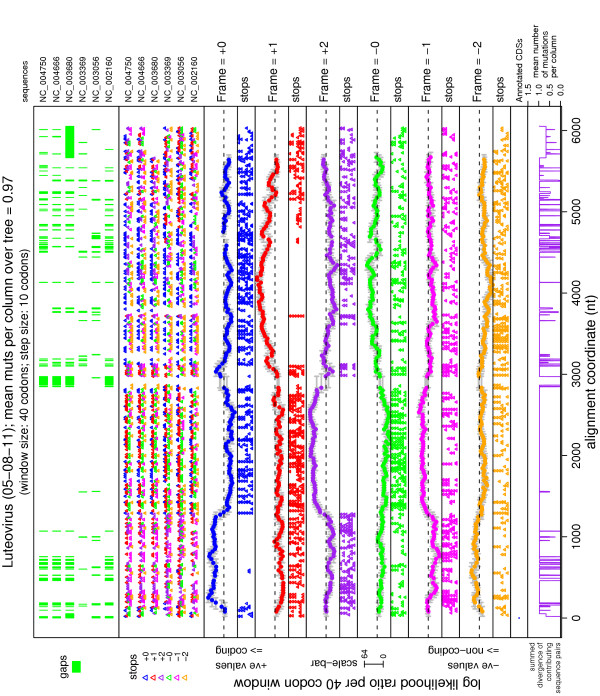
**Six-frame sliding window plot**. Example output plot for the 'Six-frame sliding window plots' option (same sequences as in Figure 1). This is a plot of the ∑_tree _log (LR) statistic calculated in a sliding window along the alignment in each of the six possible read-frames. In each window, the null model is that 'only the known CDS(s) are coding' while the alternate model is that 'both the window and the known CDS(s) are coding'. Panel 1 shows the positions of alignment gaps in each of the input sequences (labelled at right), while panel 2 shows the positions of stop codons in each of the six possible read-frames in each of the input sequences. Panel 3 shows the ∑_tree _log (LR) statistic in each window in the +0 frame (relative to reference sequence nt 1). The width of each window is indicated by horizontal grey lines (if the reference sequence contains alignment gaps within the window, then the window will appear enlarged in alignment coordinates). The horizontal dashed line is at zero. Panel 4 shows the positions of stop codons in the +0 frame in all the input sequences (same order as in panel 1). Panels 5, 7, 9, 11 and 13 show the same information as panel 3, but for the +1, +2, -0, -1 and -2 frames, respectively. Similarly, panels 6, 8, 10, 12 and 14 show the same information as panel 4, but for the +1, +2, -0, -1 and -2 frames, respectively. Panel 15 shows the known CDSs (here none were entered). Panel 16 shows the summed mean sequence divergence (mutations per nt) at each alignment position (see caption to Figure 1). (See website for more details.) Extended regions of positive signal in panels 3, 5, 7, 9, 11 and 13 indicate potential CDSs (i.e. other than those identified in the null model). In this particular plot, no known CDS(s) were entered, i.e. the null model is that the whole genome is non-coding. Hence the actual Luteovirus CDSs have clear positive signals. Note that several of the reverse read-frames show a false positive signal when they are in the -2 frame relative to a forward read-frame CDS (see website for details).

Sometimes a CDS may be entered through a stop codon read-through, ribosomal frameshift, or splicing event rather than with an AUG codon. The 'Find and test all non-annotated ORFs' option (above) may not be the best way to locate such CDSs, since such CDSs generally commence part-way through an ORF. The 'Six-frame sliding window plots' option calculates the ∑_tree _log(LR) statistic in a window sliding along the alignment in all six reading frames (Figure 2). The user may select the window and step sizes. Extended regions of positive signal may indicate potential new CDSs, especially where there is an absence of stop codons across the alignment.

## Results and discussion

### Sensitivity and selectivity

The software has been previously tested on simulated data and on overlapping CDSs in the Hepatitis B Virus and *Escherichia coli *genomes [4]. In a further test on 14 virus alignments, all 37 known CDSs were detected (including five examples of overlapping CDSs completely contained within other CDSs, and 20 CDSs that partially overlap other CDSs). Conversely, the false positive rate for all non-coding ORFs of at least 40 codons was 0.06 (-2 frame overlaps excluded; see website for details). In addition, all the false positives had very low MLOGD scores (outside the range observed for the known CDSs).

Further tests showed that, for alignments with just 20 mutations overall (i.e. ∑_tree _Λ = 20; e.g. a pairwise comparison of two 100 nt sequences with a mean divergence of 0.2 mutations per nt), MLOGD can discriminate non-overlapping CDSs from non-coding ORFs with a typical accuracy of up to 98%, and can detect CDSs overlapping known CDSs with a typical accuracy of 90% (see website for details). In general usage, ∑_tree _Λ is often much greater than 20, with correspondingly lower predicted error rates.

### On-line virus database

A database of results for 640 virus sequence alignments is available on the website. The database contains multiple sequence alignments, phylogenetic trees, positions of known CDSs, six-frame sliding window plots, statistics and plots for the annotated CDSs, and statistics and plots for all non-annotated start-stop ORFs in the reference sequences of at least 40 codons in length.

## Conclusion

We have presented (a) a new tool for locating and analysing CDSs in virus alignments, and (b) an on-line database of results in 640 virus alignments. Besides the easy-to-use website and comprehensive output, the main advantage of MLOGD over other gene-finding software is that MLOGD explicitly takes into account the possibility of overlapping genes – common in viruses. For example, for the Hepatitis B, Avian Hepatitis B, Polerovirus, Luteovirus and Human Immunodeficiency Virus 1 genomes (compact genomes with relatively high fractions of overlapping CDSs), MLOGD successfully finds all 28 known CDSs, while GeneMark only finds 17 (VIOLIN database, [7]). We have extensively tested the sensitivity of MLOGD and shown it to be more sensitive than other methods for detecting overlapping CDSs [4].

MLOGD can, of course, also be used for cellular organisms. Partially overlapping CDSs are fairly common in prokaryotes, but it is not clear what fraction of overlaps are functionally constrained. Many appear to be the result of the loss of a stop codon, allowing one CDS to run into an adjacent CDS [8]. Others may be involved in regulatory mechanisms [9]. Similarly, many potential ribosomal frameshift sites – leading to overlapping CDSs – have been identified in cellular organisms [10], as well as viral genomes. MLOGD is a valuable tool for analysing the magnitude of functional constraints on such overlaps, with implications for the annotation of putative frameshift sites, and the evolution of overlapping genes in viruses and in prokaryotes.

## Availability and requirements

The MLOGD software and virus database are available at  (see also Additional file 1). Sequences may be entered into the web-interface or the software (C++ programmes, C-shell scripts; distributed under the GNU General Public License) may be downloaded and used locally. To install the software locally, the publicly available packages EMBOSS [11] and R [12] must also be installed. The programme codaln [13] is recommended for aligning the input sequences. Run-time and resource-use scale approximately linearly with the number of sequences and the length of the input alignment. On a Pentium 4 2.8 GHz processor, analysing a 900 nt ORF takes ~3 s for a five-sequence alignment, while running six-frame sliding window plots (default window sizes) for a 10000 nt region takes ~300 s.

## Authors' contributions

AEF: algorithms, programming, website, documentation, manuscript. CMB: intellectual direction, especially with respect to biological background.

## Supplementary Material

Additional File 1 Archive of the source code. The file sup1.TGZ is an archive of the source code for the current version of MLOGD. Unpack it with tar xvfz supl.TGZ; then see the README file in the MLOGD directory. Click here for file
