# Supplementary material for: Detecting overlapping coding sequences in virus genomes
Source: BMC Bioinformatics. 2006 Feb 16;7:75. doi: 10.1186/1471-2105-7-75 (PMC1395342; doi:10.1186/1471-2105-7-75)
Supplement: Additional File 1 — Archive of the source code. The file sup1.TGZ is an archive of the source code for the current version of MLOGD. Unpack it with tar xvfz supl.TGZ; then see the README file in the MLOGD directory. [file 1471-2105-7-75-S1.TGZ › MLOGD/FORM/plot.mlenuc.html]

 
MLOGD: Notes


**Notes on the 'Nucleotide-by-nucleotide'
plot:**  
  
This is a nucleotide-by-nucleotide plot of the likelihood ratio
statistic for each reference - non-reference sequence pair and also
summed over the phylogenetic tree. Gaps, and stop and start codons for
each sequence, are also annotated on the plot. The six panels show the
following information:

1. This panel displays the raw likelihood ratio scores at each
   position in the alignment. There is a seperate track for each
   reference - non-reference sequence pair (labelled at the right).
   Gaps, and stop codons in each of the null and alternate model
   annotated CDSs, for each sequence, are marked on the appropriate
   tracks.  
     
   - This panel displays the raw likelihood ratio scores, summed over
     the phylogenetic tree (i.e. the input list of sequence pairs; details), at each column position in the input
     alignment.  
       
     - This panel displays the running mean (i.e. sliding window mean)
       likelihood ratio scores at each position in the alignment, for each
       reference - non-reference sequence pair. The sliding window width
       is annotated on the plot, and can be changed via the 'Redraw plot'
       link.  
         
       - This panel displays the running mean of the phylogenetically
         summed likelihood ratio scores (i.e. running mean of panel
         2).  
           
         - This panel shows the input Query, or alternate model, CDS(s)
           (red bars) and the input Known, or null model, CDS(s) (blue
           bars).  
             
           - This panel shows the phylogenetic sum of sequence divergences
             (mean number of mutations per nucleotide) for the sequence pairs
             that contribute to the likelihood ratio sum at each position in the
             alignment. In any particular column, some sequences may be omitted
             from the likelihood ratio calculations due to gaps or stop to
             non-stop transitions. Statistics in regions with lower summed
             divergence (i.e. partially gapped regions) have a lower
             signal-to-noise ratio.

**Notes:**

- Statistics are only shown within the query region.- For panel 4, any columns with gaps (or ambiguous nt codes), in
    any sequence in the input list of sequence pairs, are omitted. Such
    columns are omitted before taking the running mean. Thus where gaps
    occur a, for example, 21 nt window includes a total of 21 columns
    taken from either side of the gap but none from within the gap. On
    the 'Redraw plots' page, you may choose to extend the plot into
    partially gapped regions, provided the summed divergence of the
    contributing sequence pairs in the region is greater than some
    user-defined threshold value (details).- In general, you shouldn't have any null or alternate model
      stops on the reference sequence track. If there are any, then this
      means that the 'Known CDSs' or 'Query CDSs' (respectively) that you
      inputted are not in fact ORFs. I.e. you have probably misannotated
      your CDSs.- In general, you wouldn't expect to have null model stop codons
        in any of the sequences. The null model represents known CDSs,
        which are typically conserved across an alignment. Stops in some
        sequences near the ends of CDSs are not too unusual and indicate that
        the CDS terminates early in some sequences. However, if some
        sequences have many null model stops, then this may indicate a CDS
        annotation problem.- If there are no alternate model stops in the non-reference
          sequences, or any stops are located close to the 'Query CDS'
          end-point, then this indicates that the 'Query CDS' is conserved as
          an ORF across the alignment. For a long ORF, this in itself may be
          strong evidence that the ORF is a CDS. On the other hand, if
          there are many alternate model stops, then this is evidence against
          the ORF being a CDS, at least in some sequences.- Note that alignment problems may cause a non-reference sequence
            codon to be aligned out-of-frame to a reference sequence codon.
            This may occassionally result in an out-of-frame non-reference
            sequence stop codon being incorrectly annotated on the plot. This
            can be avoided by keeping gaps in groups of three within the null
            and alternate model CDSs (see also this note). Therefore, if you have an isolated
            stop codon in what otherwise appears to be a long conserved ORF, you
            should check that it is not the result of a local alignment problem.- Note also that in places where the reference sequence contains
              alignment gaps, there is no frame information for the non-reference
              sequences. As far as calculation of statistics is concerned, all
              such regions are omitted. However for the stop and start codon
              annotation, any non-reference sequence stops or starts within
              reference sequence gaps will be missed.
 
