# Supplementary material for: Detecting overlapping coding sequences in virus genomes
Source: BMC Bioinformatics. 2006 Feb 16;7:75. doi: 10.1186/1471-2105-7-75 (PMC1395342; doi:10.1186/1471-2105-7-75)
Supplement: Additional File 1 — Archive of the source code. The file sup1.TGZ is an archive of the source code for the current version of MLOGD. Unpack it with tar xvfz supl.TGZ; then see the README file in the MLOGD directory. [file 1471-2105-7-75-S1.TGZ › MLOGD/FORM/onequery.html]

 
MLOGD: Notes


**Notes on query CDSs:**  
  
You should only test one putative CDS at a time. This is because, for
each pair of input sequences, the evolutionary time parameter *t*
is fitted to the observed pairwise sequence divergence (i.e. the mean
number of mutations per nucleotide) for each of the null and alternate
models. Suppose you have three query CDSs, and one of these is a true
CDS while the other two are non-coding. If you input all three query
CDSs as your alterate model, then the alternate model *t*
parameter estimate will be too high, because the mutation rate in the
two non-coding ORFs will be higher than you would expect for true
CDSs, which is what the alternate model is assuming.  
  
If the query CDS is disjoint (e.g. due to splicing, or at two ends of
a circular genome) you can use, for example, the notation:

```
join(99..200,299..400)
```

(Make sure there are no blank spaces
in the line.) Use a similar format to describe CDSs containing
ribosomal frameshifts.  
  
If you enter a query CDS that is equal to one of the input 'Known
CDS(s)', then the programme will typically return a negative result,
implying that the alternate model (i.e. that the query CDS is coding)
is incorrect. This just means that the CDS in question is not coding
'twice'.
 
