# Supplementary material for: Detecting overlapping coding sequences in virus genomes
Source: BMC Bioinformatics. 2006 Feb 16;7:75. doi: 10.1186/1471-2105-7-75 (PMC1395342; doi:10.1186/1471-2105-7-75)
Supplement: Additional File 1 — Archive of the source code. The file sup1.TGZ is an archive of the source code for the current version of MLOGD. Unpack it with tar xvfz supl.TGZ; then see the README file in the MLOGD directory. [file 1471-2105-7-75-S1.TGZ › MLOGD/FORM/cols.dat.html]

 
MLOGD: Notes


**Description of statistics on MLOGD results
page:**  
  

1. **non-reference name:** Non-reference sequence name. Each table
   row lists statistics for a reference sequence versus non-reference
   sequence pairwise sequence comparison.  
     
   - **total # nt:** Total number of non-reference sequence
     nucleotides that align with the reference sequence within the query
     nucleotide range. I.e. number of alignment columns which are not
     gapped, or ambiguous nt codes, in either the reference or
     non-reference sequence.  
       
     - **total # muts:** Total number of point nucleotide
       differences between the non-reference sequence and the reference
       sequence, within the query nucleotide range.  
         
       - **divergence (muts / nt):** Pairwise reference sequence to
         non-reference sequence divergence (mean number of point differences
         per nucleotide). (I.e. all nucleotide positions mutation fraction.)
         Equals column 3 / column 2.   
           
         - **# nt used:** Number of nucleotides used to calculate the
           log likelihood scores. This will depend on the nucleotide range
           selected on the initial MLOGD server page, and will be reduced if
           any nucleotide positions are omitted due to gaps or ambiguous nt
           codes (in either reference or non-reference sequence), or stop to
           non-stop transitions (in either the null or alternate
           models).  
             
           - **MLOGD ln(LR) / nt:** The sum of the null versus alternate
             model log likelihood ratios over the whole pairwise sequence
             comparison, divided by the number of nucleotides used.  
               
             - **frac null muts:** Fraction of the aligned codons, within the
               null model CDSs, that are identical between the two sequences.  
                 
               - **frac syn muts:** Fraction of the aligned codons, within the
                 null model CDSs, that are different between the two sequences but
                 code for identical amino acids. (I.e. synonymous codon mutation
                 fraction.)  
                   
                 - **frac nonsyn muts:** Fraction of the aligned codons, within
                   the null model CDSs, that are different between the two sequences and
                   code for different amino acids. (I.e. nonsynonymous codon mutation
                   fraction.)  
                     
                   - **frac N1 muts:** Fraction of the 1st codon position
                     nucleotides (e.g. the C in CAG *gln*), within the null model
                     CDSs, that are different between the two sequences. (I.e. 1st codon
                     position mutation fraction.)  
                       
                     - **frac N2 muts:** Fraction of the 2nd codon position
                       nucleotides (e.g. the A in CAG *gln*), within the null model
                       CDSs, that are different between the two sequences. (I.e. 2nd codon
                       position mutation fraction.)  
                         
                       - **frac N3 muts:** Fraction of the 3rd codon position
                         nucleotides (e.g. the G in CAG *gln*), within the null model
                         CDSs, that are different between the two sequences. (I.e. 3rd codon
                         position mutation fraction.)

**Notes:**

- Columns 7 + 8 + 9 sum to one, unless the null model has no
  CDSs annotated, in which case all three values are zero.- If the entire alignment is coding in the null model, then the
    average of columns 10, 11 and 12 is the pairwise sequence
    divergence, column 4. If the null model has no CDSs annotated then
    columns 10, 11 and 12 are zero.- Multiplying column 6 by column 5 gives the log likelihood ratio
      for the whole pairwise sequence comparison. Positive values favour
      the alternate model, while negative values favour the null model.
 
