# Supplementary material for: Detecting overlapping coding sequences in virus genomes
Source: BMC Bioinformatics. 2006 Feb 16;7:75. doi: 10.1186/1471-2105-7-75 (PMC1395342; doi:10.1186/1471-2105-7-75)
Supplement: Additional File 1 — Archive of the source code. The file sup1.TGZ is an archive of the source code for the current version of MLOGD. Unpack it with tar xvfz supl.TGZ; then see the README file in the MLOGD directory. [file 1471-2105-7-75-S1.TGZ › MLOGD/FORM/aln_note.html]

 
MLOGD: Notes


**Note on sequence alignment quality:**  
  
Knowing the correct reading-frame is very important for the
statistical mutation model. When estimating the probability that a
given nucleotide in a CDS will mutate, the software needs to know
which codon the nucleotide is in, and its position within that codon.
The reference sequence CDS files are used to define all the
reading-frames. Every coding nucleotide in the reference sequence is
assigned a codon position (1st, 2nd or 3rd) and read-direction
(forward or reverse). Nucleotides in the non-reference sequences are
identified purely on the basis of the nucleotides in the reference
sequence with which they align. (See also note on reference sequence quality.)  
  
In order to maintain the correct reading-frame in the different
sequences, it is important that gaps in the alignment occur in groups
of three within coding sequences. A useful programme for doing this
is 
code2aln (Stocsits 2003). This is a sequence alignment
programme that tries to keep gaps in groups of three in CDSs and
smoothly joins coding regions onto non-coding regions. A newer version
- called 
codaln - is also available (Stocsits et al 2005).  
  
Provided you don't have too many sequences, you can enter your
sequences at this site, which uses
code2aln to realign the sequences. If you have purely coding
sequences you can of course use e.g. CLUSTALW
on the translated (amino acid) sequences.  
  
Note also that in places where the reference sequence contains
alignment gaps, there is no frame information for the non-reference
sequences. As far as calculation of statistics is concerned, all such
regions are omitted. However, for the stop and start codon annotation
on some of the output plots, any non-reference sequence stops or
starts within reference sequence gaps will be missed.  
  
If your alternate model is an ORF overlapping and entirely contained
within a known CDS, then alignment on the known CDS amino acid
sequence will ensure that gaps within the putative CDS occur in groups
of three. If, however, the putative CDS is partially or entirely
contained within a region that is non-coding in the null model, then
it will be harder to ensure that the alignment programme maintains
gaps within the putative CDS in groups of three. You can improve the
chances that it does so, by annotating the putative CDSs as well as
known CDSs on the alignment server page.  
  
**References:**

- Roman R. Stocsits, 2003, Nucleic Acid Sequence Alignments of
  Partly Coding Regions, PhD thesis, University of Vienna.- Roman R. Stocsits, Ivo L. Hofacker, Claudia Fried, Peter
    F. Stadler, 2005, Multiple Sequence Alignments of Partially Coding
    Nucleic Acid Sequences, *BMC Bioinformatics*, **6**, 160.
 
