# Supplementary material for: Detecting overlapping coding sequences in virus genomes
Source: BMC Bioinformatics. 2006 Feb 16;7:75. doi: 10.1186/1471-2105-7-75 (PMC1395342; doi:10.1186/1471-2105-7-75)
Supplement: Additional File 1 — Archive of the source code. The file sup1.TGZ is an archive of the source code for the current version of MLOGD. Unpack it with tar xvfz supl.TGZ; then see the README file in the MLOGD directory. [file 1471-2105-7-75-S1.TGZ › MLOGD/FORM/ntrange.html]

 
MLOGD: Notes


**Notes on nucleotide range:**  
  
You can compare the alternate model against the null model over the
whole input alignment if you like (e.g. a virus genome alignment).
However, if the query ORF is only a relatively small region in the
genome, then you will probably get better statistics if you limit the
nucleotide range investigated to just encompass the query ORF.
Outside the query ORF, the null and alternate models are essentially
identical (although the likelihood scores may differ slightly due to
different fitted *t* values - more-or-less evolutionary time -
for the two models). Therefore the likelihood ratio for each
nucleotide in this region will be uninformative. These uninformative
values can make interpretation of the final summed-over-sequence
statistics confusing, and will 'dilute' the coding signature (if it
exists) of the query ORF.  
  
However, if your query ORF is fairly short (e.g. < 40 codons) then you
may want to take a larger nucleotide range, encompassing the query
ORF. This is because, for short regions, there may not be enough
nucleotide mutations to accurately determine *t* for either the
null or alternate models.  
  
Since alignments may be poorer at the ends of a reference sequence ORF
(e.g. if the ORF utilizes a later start or earlier stop codon in other
sequences), sometimes you may want to set the nucleotide range to a
smaller region than the reference sequence ORF. In this case, we
suggest that first you enter the whole reference sequence ORF. Then
you can use the nucleotide-by-nucleotide plot on the results page to
check for stops in the non-reference sequences, and reduce the
nucleotide range accordingly in a second run.  
  
  
**Dependence on 'Operating mode':**  
  
**Test input query CDSs:** Here you can use any of the three
nucleotide range options. The recommended option is 'Only use nt
within query CDS(s)', unless the query CDS is less than about 40
codons (depending on the number of sequences and their total
divergence) in which case you may want to use the 'Use a given
nucleotide range' option to specify a region of about 40-100 codons
length, encompassing the query ORF. The models will be fitted, and
statistics calculated only within the query CDS(s) (which may be
disjoint).  
  
Note that if the 'Use a given nucleotide range' option is selected,
and the first number is greater than the second, then the two numbers
will be swapped round. I.e. you can't use this option to cross the
'boundary' on a circular genome. Instead you can use the 'Only use nt
within query CDS(s)' option with a 'Query CDS' such as, for example,
'join(2000..3000,1..1000)'.  
  
**Find and test all non-annotated ORFs:** Whichever of the three
options is selected, the region used for calculating statistics for
each located ORF is just the ORF itself. Instead the nucleotide range
options are used to select the range for ORF detection. If the 'Use
whole sequence' option is selected, then the whole sequence will be
used for ORF detection. If the 'Use a given nucleotide range' option
is selected, then only ORFs within that range will be used. If the
'Only use nt within query CDS(s)' option is selected, then it will
default to the 'Use whole sequence' option.  
  
**Six-frame sliding window plots:** Whichever of the three options
is selected, the region used for calculating statistics for each
window is just the window itself. Instead the nucleotide range
options are used to select the range for which six-frame sliding
window plots will be produced. If the 'Use whole sequence' option is
selected, then six-frame sliding window plots will be produced for the
whole sequence. If the 'Use a given nucleotide range' option is
selected, then six-frame sliding window plots will be produced only
for the given region. If the 'Only use nt within query CDS(s)' option
is selected, then it will default to the 'Use whole sequence'
option.  
  
 
