# Supplementary material for: Detecting overlapping coding sequences in virus genomes
Source: BMC Bioinformatics. 2006 Feb 16;7:75. doi: 10.1186/1471-2105-7-75 (PMC1395342; doi:10.1186/1471-2105-7-75)
Supplement: Additional File 1 — Archive of the source code. The file sup1.TGZ is an archive of the source code for the current version of MLOGD. Unpack it with tar xvfz supl.TGZ; then see the README file in the MLOGD directory. [file 1471-2105-7-75-S1.TGZ › MLOGD/FORM/mode.html]

 
MLOGD: Notes


**MLOGD operating modes:**  
  


---

**Test input query CDSs:**  
  
Here MLOGD calculates the likelihood ratio for the
alternative model versus the null model in a given region. The null
model is that the only CDSs (if any) within the region, are the CDSs
entered in the 'Known CDSs' box. The alternative model is that the
ORFs entered in the 'Query CDSs' box are also coding. Thus
MLOGD tests the double-coding versus single-coding model
where the query CDSs overlap a known CDS, and the single-coding versus
non-coding model elsewhere. In general, you should only enter and test one
putative CDS in the 'Query CDSs' box at a time.  
  
On the results page you will get a lot of statistics and also the
option to run Monte Carlo simulations (useful for estimating error
bars on the results) and to redo the windowing on the
nucleotide-by-nucleotide plot. When using the 'Find and test all
non-annotated ORFs' or the 'Six-frame sliding window plots' options
below, you won't get the options to do the Monte Carlo simulations
(which would take a long time to run for all ORFs/windows). If you
find some interesting ORFs/windows, then you can of course go back
with the 'Test input query CDSs' option, one ORF/window at a time, to
get these extra options.  
  

---

**Find and test all non-annotated ORFs:**   
  
Here MLOGD looks for all ORFs longer than the specified
minimum length (except those entered in the 'Known CDSs' box). For
each ORF, MLOGD calculates the likelihood ratio for the
alternative model versus the null model. The null model is that the
only CDSs (if any) within the query ORF, are the CDSs entered in the
'Known CDSs' box. The alternative model is that the new ORF is also
coding.  
  
Note that one of the new ORFs may be nearly-identical to one of the
'Known CDSs' (e.g. if you included the stop codons in the 'Known CDSs'
annotation, so that the new ORF is not recognized as being identical to
one of the 'Known CDSs'). In this case, in the alternate model, the
amino acid and codon substitution weights are actually applied twice
for the same CDS. Generally you should ignore the results for such
query ORFs.  
  
If you select 'start-stop' ORFs, then MLOGD will calculate
the alternative versus null model likelihood ratio for each ORF that
starts with AUG and ends with UAG, UGA or UAA. If you select
'stop-stop' ORFs then MLOGD will calculate the alternative
versus null model likelihood ratio for ORFs running from immediately
after a UAG, UGA or UAA to the next in-frame UAG, UGA or UAA.
  
  

---

**Six-frame sliding window plots:**  
  
Sometimes an ORF may be entered through a stop codon read-through,
ribosomal frameshift, or splicing event rather than with an AUG codon.
However, just testing the likelihood ratio score for all stop-stop
ORFs (using the 'Find and test all non-annotated ORFs' option above)
may not be the best way to locate such ORFs, since the end of the
'stop-stop' ORF will follow the alternative model, while the beginning
may follow the null model. You don't necessarily know where the
changeover takes place. In contrast, in the 'Six-frame sliding window
plots' mode, MLOGD will calculate and plot the likelihood
ratio for *N*-codon windows, moving along the reference sequence
in *M*-codon steps. Plot are produced for all three forward and
three backward read-frames. You need to choose the sliding window
size *N* to compromise between low nt resolution (for large
*N*) and noisy likelihood ratio statistics (for small
*N*).  
  

---

 
