# Supplementary material for: Detecting overlapping coding sequences in virus genomes
Source: BMC Bioinformatics. 2006 Feb 16;7:75. doi: 10.1186/1471-2105-7-75 (PMC1395342; doi:10.1186/1471-2105-7-75)
Supplement: Additional File 1 — Archive of the source code. The file sup1.TGZ is an archive of the source code for the current version of MLOGD. Unpack it with tar xvfz supl.TGZ; then see the README file in the MLOGD directory. [file 1471-2105-7-75-S1.TGZ › MLOGD/FORM/cols.mcprobs.html]

 
MLOGD: Notes


**Description of statistics on Monte Carlo
results page:**  
  

1. **non-reference name:** Non-reference sequence name. Each table
   row lists statistics for a reference sequence versus non-reference
   sequence pairwise sequence comparison.  
     
   - **# nt used:** Number of nucleotides used to calculate the
     log likelihood scores. This will depend on the nucleotide range
     selected on the initial MLOGD server page, and will be reduced if
     any nucleotide positions are omitted due to gaps or ambiguous nt
     codes (in either sequence), or stop to non-stop transitions (in
     either the null or alternate models).  
       
     - **divergence (mean muts per nt):** Pairwise sequence
       divergence (mean number of point differences per nucleotide).
       (I.e. all nucleotide positions mutation fraction.)  
         
       - **MLOGD ln(LR) per nt:** The sum of the null versus alternate
         model log likelihood ratios over the whole pairwise sequence
         comparison, divided by the number of nucleotides used.  
           
         - **frac syn muts:** Fraction of the aligned codons, within the
           null model CDSs, that are different between the two sequences but
           code for identical amino acids. (I.e. synonymous codon mutation
           fraction.)  
             
           - **frac nonsyn muts:** Fraction of the aligned codons, within
             the null model CDSs, that are different between the two sequences and
             code for different amino acids. (I.e. nonsynonymous codon mutation
             fraction.)  
               
             - **frac N1 muts:** Fraction of the 1st codon position
               nucleotides (e.g. the C in CAG *gln*), within the null model
               CDSs, that are different between the two sequences. (I.e. 1st codon
               position mutation fraction.)  
                 
               - **frac N2 muts:** Fraction of the 2nd codon position
                 nucleotides (e.g. the A in CAG *gln*), within the null model
                 CDSs, that are different between the two sequences. (I.e. 2nd codon
                 position mutation fraction.)  
                   
                 - **frac N3 muts:** Fraction of the 3rd codon position
                   nucleotides (e.g. the G in CAG *gln*), within the null model
                   CDSs, that are different between the two sequences. (I.e. 3rd codon
                   position mutation fraction.)  
                     
                   - **MLOGD ln(LR):** Null versus alternate model log likelihood
                     ratio for the MLOGD statistic, estimated by comparing the
                     observed value with simulated distributions.  
                       
                     - **syn ln(LR):** Null versus alternate model log likelihood
                       ratio for the 'frac syn muts' statistic, estimated by comparing the
                       observed value with simulated distributions.  
                         
                       - **nonsyn ln(LR):** Null versus alternate model log likelihood
                         ratio for the 'frac nonsyn muts' statistic, estimated by comparing the
                         observed value with simulated distributions.  
                           
                         - **N1 ln(LR):** Null versus alternate model log likelihood
                           ratio for the 'frac N1 muts' statistic, estimated by comparing the observed
                           value with simulated distributions.  
                             
                           - **N2 ln(LR):** Null versus alternate model log likelihood
                             ratio for the 'frac N2 muts' statistic, estimated by comparing the observed
                             value with simulated distributions.  
                               
                             - **N3 ln(LR):** Null versus alternate model log likelihood
                               ratio for the 'frac N3 muts' statistic, estimated by comparing the observed
                               value with simulated distributions.

**Notes:**

- If the null model is non-coding (no 'Known CDSs' entered) then
  columns 5-9 will all be 0.00 and columns 11-15 will all be 99999.0.- For columns 10-15, see Firth A. E., Brown C. M., 2005,
    *Bioinformatics*, **21**, 282-92 and Section 2.5 of the
    Supplementary Material pdf file
    (1.0MB), ps file (0.9MB) for details of
    this calculation.- If the entire nucleotide range selected on the initial MLOGD
      server page is coding in the null model, then the average of columns
      7, 8 and 9 should equal column 2, while the average of columns 5 and
      6 would generally be larger since these statistics count mutated
      codons rather than single nucleotides.- Multiplying columns 2 and 4 gives the null versus alternate
        model log likelihood ratio summed over the whole pairwise sequence
        comparison. In principal this is the same statistic as in column
        10. However, there will generally be significant disagreement due
        to the approximations made in the calculation of the column 10
        statistic (details).
 
