# Supplementary material for: Detecting overlapping coding sequences in virus genomes
Source: BMC Bioinformatics. 2006 Feb 16;7:75. doi: 10.1186/1471-2105-7-75 (PMC1395342; doi:10.1186/1471-2105-7-75)
Supplement: Additional File 1 — Archive of the source code. The file sup1.TGZ is an archive of the source code for the current version of MLOGD. Unpack it with tar xvfz supl.TGZ; then see the README file in the MLOGD directory. [file 1471-2105-7-75-S1.TGZ › MLOGD/FORM/mcprobs_note.html]

 
MLOGD: Notes


**Note on calculation of likelihood ratios using
Monte Carlo simulations:**  
  
The statistics in columns 4-9 of the table at the bottom of the Monte
Carlo simulations results page are determined for each reference -
non-reference sequence pair in the input alignment. We wish to use
these statistic to try to determine which of the null model or
alternate model CDSs provide a better model for the pattern of
mutations observed in the input alignment.  
  
Column 4 (the MLOGD likelihood ratio score) may be used directly as
such a classifier: values less than zero support the null model, while
values greater than zero support the alternate model. We recommend
using this score. However, in Firth & Brown, 2005,
*Bioinformatics*, **21**, 282-92, we also investigated using
the other scores: 1st/2nd/3rd codon position mutation fractions and
synonymous/nonsynonymous codon mutation fractions. In order to
interpret these scores, we resort to simulations to find out what
range of values we would expect to observe for each of the null and
alternate models.  
  
Ideally one would estimate the probability of obtaining each observed
score, under each of the null and alternate models, directly from the
simulations. In practice it would require a very large number of
simulations to estimate the near-zero probabilities that occur
whenever the observed score is incompatible with one or other of the
models (e.g. if the alternate model is actually correct, then trying
to estimate the probability of the observed score occuring under the
null model might be very difficult). Instead we assume a normal
probability distribution and use the simulations to estimate the
distribution parameters (mean and standard deviation). Using this, we
can determine the probabilities P(*x* | null model) and
P(*x* | alternate model), for each score
*x*, and hence find the null versus alternate model log
likelihood ratio. These are the scores in columns 10-15. Values less
than zero support the null model, while values greater than zero
support the alternate model.  
  
This classification scheme is simple to implement but ignores
deviations from normal distributions (e.g. columns 5-9 are constrained
to be non-negative), so columns 10-15 are not strictly speaking
likelihood ratios, but may still be used as classifiers. Note that,
even if they were true likelihood ratios, converting the likelihood
ratios to probabilities would still require Bayesian prior
probabilities on the null and alternate models.  
  
You may like to combine the 1st/2nd/3rd codon position mutation
fraction scores into one score (i.e. columns 10 + 11 + 12), and
similarly for the synonymous/nonsynonymous codon mutation fraction
scores (i.e. columns 13 + 14). Be aware, however, that
non-independence of these scores will compromise the statistics. In
particular, the 3rd codon position score is completely determined if
you know the 1st and 2nd codon position scores, so you may like to use
(2/3) \* (columns 10 + 11 + 12), instead.  
  
Full details of the above are given in Firth A. E., Brown C. M., 2005,
Detecting overlapping coding sequences with pairwise alignments,
*Bioinformatics*, **21**, 282-92 and Section 2.5 of the
Supplementary Material pdf file (1.0MB),
ps file (0.9MB).  
  
 
