# Supplementary material for: Detecting overlapping coding sequences in virus genomes
Source: BMC Bioinformatics. 2006 Feb 16;7:75. doi: 10.1186/1471-2105-7-75 (PMC1395342; doi:10.1186/1471-2105-7-75)
Supplement: Additional File 1 — Archive of the source code. The file sup1.TGZ is an archive of the source code for the current version of MLOGD. Unpack it with tar xvfz supl.TGZ; then see the README file in the MLOGD directory. [file 1471-2105-7-75-S1.TGZ › MLOGD/FORM/plot.sixframe.html]

 
MLOGD: Notes


**Notes on the MLOGD 'Six-frame' plot:**  
  
This is a plot of the MLOGD statistic calculated in a sliding window
along the alignment in each of the six possible read-frames. In each
window, the likelihood ratio that 'Both the window and the input Known
CDS(s) are coding' versus 'Only the input Known CDS(s) are coding' is
calculated and summed over the phylogenetic tree (as described here). The sixteen panels show the following
information:

1. This panel shows the positions of alignment gaps in each of the
   input sequences (labelled at right).- This panel shows the positions of stop codons in each of the six
     possible read-frames in each of the input sequences (labelled at
     right).- This panel shows the likelihood ratio score in each window in
       the +0 frame (relative to reference sequence nucleotide 1), summed
       over the input sequence pairs. The width of the window is indicated
       by the horizontal grey line. The window width is determined on the
       reference sequence, so if the reference sequence contains alignment
       gaps within the window, then the window will appear larger in
       alignment coordinates. The dashed line is at zero.- This panel shows the positions of stop codons in the +0 frame in
         all the input sequences (same order as in panel 1).- As panel 3, +1 frame.- As panel 4, +1 frame.- As panel 3, +2 frame.- As panel 4, +2 frame.- As panel 3, -0 frame.- As panel 4, -0 frame.- As panel 3, -1 frame.- As panel 4, -1 frame.- As panel 3, -2 frame.- As panel 4, -2 frame.- Input Known, or null model, CDS(s).- This panel shows the phylogenetic sum of sequence divergences
                                 (mean number of mutations per nucleotide) for the sequence pairs
                                 that contribute to the likelihood ratio sum at each position in the
                                 alignment. In any particular column, some sequences may be omitted
                                 from the likelihood ratio calculations due to gaps or stop to
                                 non-stop transitions. Statistics in regions with lower summed
                                 divergence (i.e. partially gapped regions) have a lower
                                 signal-to-noise ratio.

  
**Notes:**

- In general, you wouldn't expect to have in-frame stop codons
  within any of the annotated CDSs, since the annotated CDSs should in
  general be conserved across the alignment. A few stops near the
  ends of CDSs are not unusual and indicate that the CDS terminates
  early in some sequences. However, if there are many in-frame stop
  codons within the annotated CDSs, then this may indicate a CDS
  annotation or alignment problem.- Note that the scores in the plots have been summed over the
    input pairs file or phylogenetic tree. If a CDS is present in some,
    but not all, of the input sequences, then the pattern of mutations
    consistent with coding in some of the sequences will be diluted by
    the pattern of mutations consistent with non-coding in the other
    sequences.- Note that alignment problems may cause a non-reference sequence
      codon to be aligned out-of-frame to a reference sequence codon.
      This may occassionally result in an out-of-frame non-reference
      sequence stop codon being incorrectly annotated on the plot. This
      can be avoided by keeping gaps in groups of three within CDSs (see
      also this note). Therefore, if you have
      an isolated stop codon in what otherwise appears to be a long
      conserved ORF, you should check that it is not the result of a local
      alignment problem.- Note also that in places where the reference sequence contains
        alignment gaps, there is no frame information for the non-reference
        sequences. As far as calculation of statistics is concerned, all
        such regions are omitted. However for the stop and start codon
        annotation, any non-reference sequence stops or starts within
        reference sequence gaps will be missed.- For panels 3, 5, 7, 9, 11 and 13, the score for a particular
          window may be omitted from the plot if it is partially or total
          gapped in some or all of the sequence pairs. Scores are omitted if
          the sum, **S**, over sequence pairs of (number of nt used in
          window) x (pairwise sequence divergence) is less than some threshold
          value (details). Scores from partially
          gapped regions which are not omitted, are scaled by **S\_max** /
          **S** (details).
 
