# Supplementary material for: Detecting overlapping coding sequences in virus genomes
Source: BMC Bioinformatics. 2006 Feb 16;7:75. doi: 10.1186/1471-2105-7-75 (PMC1395342; doi:10.1186/1471-2105-7-75)
Supplement: Additional File 1 — Archive of the source code. The file sup1.TGZ is an archive of the source code for the current version of MLOGD. Unpack it with tar xvfz supl.TGZ; then see the README file in the MLOGD directory. [file 1471-2105-7-75-S1.TGZ › MLOGD/FORM/plot.mle.html]

 
MLOGD: Notes


**Notes on the MLOGD 'Likelihood ratio'
plot:**  
  
This is a plot of the MLOGD scores for each reference sequence -
non-reference sequence pair, as a function of pairwise sequence
divergence (red circles).  
  
The MLOGD score is the sum of the null versus alternate model log
likelihood ratios over the whole pairwise sequence comparison, divided
by the number of nucleotides used (i.e. column 6 of the statistics
summary table). Positive values favour the alternate model while
negative values favour the null model.  
  
The blue circle (if shown) represents the sum over the phylogenetic
tree (details). Sometimes the blue circle will
be outside the plot range (e.g. if the summed divergence over the tree
is > 0.8 mutations per column). However, the coordinates of the blue
circle are also given below the plot.  
  
Note that all these scores are *per nucleotide* likelihood
scores. A very short pairwise alignment can, by chance, have a few
particular mutations that lead to an unusually large *per
nucleotide* likelihood score. For a longer pairwise alignment, or
an alignment of many sequences, the *per nucleotide* likelihood
score is likely to be more accurate. Thus it is the total log
likelihood score, rather than the *per nucleotide* likelihood
score, that should, in the end, be used to put a value on how strongly
the input sequence data discriminates between the null and alternate
models.
 
