# Supplementary material for: Detecting overlapping coding sequences in virus genomes
Source: BMC Bioinformatics. 2006 Feb 16;7:75. doi: 10.1186/1471-2105-7-75 (PMC1395342; doi:10.1186/1471-2105-7-75)
Supplement: Additional File 1 — Archive of the source code. The file sup1.TGZ is an archive of the source code for the current version of MLOGD. Unpack it with tar xvfz supl.TGZ; then see the README file in the MLOGD directory. [file 1471-2105-7-75-S1.TGZ › MLOGD/FORM/refseq_note.html]

 
MLOGD: Notes


**Notes on reference sequence quality:**  
  
The reference sequence (used for the CDS annotation) mustn't have
sequencing-error indels within CDSs, as these will throw
the software out of read-frame and cause global problems. However
indels are tolerated in the other sequences: there'll be a local
incorrect codon identification, but no long-range problems. Bad
alignments or local (paired) frameshifts (gaps not in threes) will
mean that nucleotides within the 'mis-aligned' region may get
wrong codon position identifications - leading to wrong codon
identifications - but the problems should be local. It is up to the
user to check for alignment problems. In non-coding regions, of
course, gaps not in threes are allowed and don't cause
problems.  
  
 
