# Supplementary material for: Detecting overlapping coding sequences in virus genomes
Source: BMC Bioinformatics. 2006 Feb 16;7:75. doi: 10.1186/1471-2105-7-75 (PMC1395342; doi:10.1186/1471-2105-7-75)
Supplement: Additional File 1 — Archive of the source code. The file sup1.TGZ is an archive of the source code for the current version of MLOGD. Unpack it with tar xvfz supl.TGZ; then see the README file in the MLOGD directory. [file 1471-2105-7-75-S1.TGZ › MLOGD/FORM/tree.html]

 
MLOGD: Tree diagram


  
  
Example phylogenetic tree (for 12 Hepatitis B Virus
strains). The (unrooted) tree is used to select a list of sequence
pairs tracing round the outside of the tree. For this tree, the 12
sequence pairs would be reference - X70185, X70185 - D00329, D00329
- woodchuck, woodchuck - monkey, monkey - X75663, X75663 - X02496,
X02496 - X75664, X75664 - AB056514, AB056514 - X75665, X75665 -
AB074755, AB074755 - D50520 and D50520 - reference. Note that this
set of pairwise comparisons covers each branch of the tree precisely
twice - hence no branch is given more weight than another. In
general, the set of pairs selected in this way is not unique - e.g.
branches of the tree may be flipped into different places without
changing the phylogeny.  
  
In MLOGD, as well as calculating nucleotide-by-nucleotide MLOGD scores
for each reference sequence - non-reference sequence pair, scores are
also calculated for each of the pairs tracing round the tree, and
summed up over all pairs to give a phylogenetically-summed MLOGD
score. In fact the sum is then divided by two (since each branch is
covered twice by the above pairs) and then divided by two again (since
for each pair A - B, both A versus B and B versus A MLOGD scores are
calculated; the two scores may differ slightly due to the treatment
of gaps etc.).  
  
 
