# Supplementary material for: Detecting overlapping coding sequences in virus genomes
Source: BMC Bioinformatics. 2006 Feb 16;7:75. doi: 10.1186/1471-2105-7-75 (PMC1395342; doi:10.1186/1471-2105-7-75)
Supplement: Additional File 1 — Archive of the source code. The file sup1.TGZ is an archive of the source code for the current version of MLOGD. Unpack it with tar xvfz supl.TGZ; then see the README file in the MLOGD directory. [file 1471-2105-7-75-S1.TGZ › MLOGD/FORM/montecarlo.html]

 
MLOGD: Notes


**Notes on Monte Carlo simulations:**  
  
MLOGD provides an estimate of the probability ratio for the null model
versus the alternate model. However, it doesn't directly provide
confidence limits on this estimate. One way to estimate confidence
limits is to calculate the distribution of scores observed for a large
number of simulated alignments with the same initial reference
sequence and pairwise divergences as the input alignment. The
simultations involve applying random mutations to the reference
sequence, using the nucleotide, codon and amino acid substitution
matrices, together with either the null or alternate model CDS
annotation, to weight the mutation probabilities at each
nucleotide.  
  
On the Monte Carlo simulations page, you can

- Run simulations for each reference - non-reference sequence pair
  using either the null model or the alternate model. Typically you'd
  run about 50-100 simulations for each model, using the same pairwise
  divergence as the original reference - non-reference sequence pair.
  The width of the distribution of the MLOGD scores for the simulated
  sequence pairs can be used to estimate confidence limits on the
  MLOGD score for the original reference - non-reference sequence pair.- Run simulations for a range of pairwise divergences using either
    the null model or the alternate model. The MLOGD scores for the
    reference - simulated sequence pairs can be used to plot the general
    distribution of the MLOGD score as a function of pairwise sequence
    divergence for the particular reference sequence, CDS annotation and
    query ORF.

In addition, on the Monte Carlo simulations results page, you will
get plots for the N123 (i.e. 1st/2nd/3rd codon position mutation
fraction) and NsNn (i.e. synonymous/nonsynonymous mutation fraction)
statistics introduced in Firth A. E., Brown C. M., 2005, Detecting
overlapping coding sequences with pairwise alignments,
*Bioinformatics*, **21**, 282-92, together with null model
versus alternate model likelihood ratios calculated by comparing the
observed statistics with the distribution of null and alternate model
scores obtained from the simulations.
 
