# Supplementary material for: Detecting overlapping coding sequences in virus genomes
Source: BMC Bioinformatics. 2006 Feb 16;7:75. doi: 10.1186/1471-2105-7-75 (PMC1395342; doi:10.1186/1471-2105-7-75)
Supplement: Additional File 1 — Archive of the source code. The file sup1.TGZ is an archive of the source code for the current version of MLOGD. Unpack it with tar xvfz supl.TGZ; then see the README file in the MLOGD directory. [file 1471-2105-7-75-S1.TGZ › MLOGD/FORM/threshold3.html]

 
MLOGD: Notes


**Note on threshold for including windows in
Six-frame plot:**  
  
In the 'Six-frame' plot, the score for a particular window may
be omitted from the plot if it is partially or total gapped in some or
all of the sequence pairs. Specifically, for each window, the sum,
**S**, over sequence pairs of (number of nt used in window) x
(pairwise sequence divergence) is calculated. Here, the sequence
divergence of each pair is the mean number of mutations per nucleotide
for the whole sequence (rather than just in the window), and the
number of nt used is generally equal to the window length, but may be
reduced for gaps, ambiguous nt codes or stop to non-stop transitions.
This is basically a measure of how much data is available in each
window to calculate the likelihood ratio statistic; lower values
correspond to lower signal-to-noise.  
  
If the sum **S** is at least (threshold) x **S\_max** then the
likelihood ratio statistic for this window is included in the
six-frame plot. Otherwise the window is omitted. The default
threshold is 0.75. On the 'Redraw plots' page, you have the option to
change this threshold value (minimum 0.01, maximum 0.99).
**S\_max**, the maximum possible value of **S**, is basically the
window length (in nt) times the total pairwise divergence summed over
all sequence pairs.  
  
Before plotting, each window's likelihood score is scaled by
**S\_max** / **S**. This scaling is applied so that you don't get
dips in the six-frame plot just due to some sequences being gapped in
some regions. Be aware, however, that in partially gapped regions the
signal-to-noise ratio will be lower compared with neighbouring
ungapped regions.  
  
The original unscaled likelihood scores can be found by following the
link to 'raw plot data' -> 'Likelihood ratios' -> column 3. These
scores represent the actual likelihood ratio statistics for each
window, based on the information present in that window - i.e. if some
sequences are partially gapped (or have ambiguous nt codes) then there
is less information so, all other things being equal, the likelihood
ratios are lower.  
  
 
