# Supplementary material for: Detecting overlapping coding sequences in virus genomes
Source: BMC Bioinformatics. 2006 Feb 16;7:75. doi: 10.1186/1471-2105-7-75 (PMC1395342; doi:10.1186/1471-2105-7-75)
Supplement: Additional File 1 — Archive of the source code. The file sup1.TGZ is an archive of the source code for the current version of MLOGD. Unpack it with tar xvfz supl.TGZ; then see the README file in the MLOGD directory. [file 1471-2105-7-75-S1.TGZ › MLOGD/FORM/egplots.html]

 
MLOGD: Notes


**Example plots for the different running modes:**  

- 'Test input query CDSs' and 'Find and test all non-annotated ORFs':
  - Likelihood ratio plot (description)- Nucleotide-by-nucleotide plot (description)- Zoomed-in nucleotide-by-nucleotide
        plot (description)- Monte Carlo simulations plot (description)  
  - 'Six-frame sliding window plots':
    - Six-frame plot (description)

More information on the contents of these plots is given on the
relevant results pages.  
  
  
**Example full output for the different running
modes:**  

- Test input query CDSs
  (Hepatitis B Virus - S gene taken as the Query CDS; P, C and X genes
  taken as the Known CDSs.)- Find and test all
    non-annotated ORFs (Enterovirus with single long polyprotein
    CDS annotated.)- Six-frame sliding window
      plots (Luteovirus with no Known CDSs annotated.)
 
