# Supplementary material for: Detecting overlapping coding sequences in virus genomes
Source: BMC Bioinformatics. 2006 Feb 16;7:75. doi: 10.1186/1471-2105-7-75 (PMC1395342; doi:10.1186/1471-2105-7-75)
Supplement: Additional File 1 — Archive of the source code. The file sup1.TGZ is an archive of the source code for the current version of MLOGD. Unpack it with tar xvfz supl.TGZ; then see the README file in the MLOGD directory. [file 1471-2105-7-75-S1.TGZ › MLOGD/FORM/threshold1.html]

 
MLOGD: Notes


**Note on threshold for including columns in
summed running mean track:**  
  
In the default 'Nucleotide-by-nucleotide' and zoomed-in
'Nucleotide-by-nucleotide' plots, the running mean of the log
likelihood scores summed over the input phylogenetic tree (i.e. the
input pairs file) skips regions that are gapped (or have ambiguous nt
codes) in any of the sequences in the pairs file. On the 'Redraw
plots' page, you have the option to extend this track into gapped
regions, provided the summed divergence of the contributing sequence
pairs in the region is >= (threshold) x (maximum divergence). (The
maximum divergence is just the summed divergence in ungapped parts of
the alignment. 0.01 <= threshold <= 0.99; default = 1.)  
  
Where some sequence pairs are omitted due to gaps (or ambiguous nt
codes), but the summed divergence is still >= (threshold) x (maximum
divergence), the log likelihood scores are scaled by (maximum
divergence) / (local divergence), before taking the running mean.
(The local divergence is just the summed divergence in that particular
column of the alignment.) This scaling is applied so that you don't
get dips in the running mean just due to some sequences being gapped.
Be aware, however, that in partially gapped regions the
signal-to-noise ratio will be lower compared with neighbouring
ungapped regions.  
  
The values in the raw summed-over-tree likelihood scores track are not
scaled by (maximum divergence) / (local divergence), because these
scores represent the actual likelihoods, based on the information in
each column - i.e. if some sequences are gapped (or have ambiguous nt
codes) then there is less information so, all other things being
equal, the likelihood ratios are lower.  
  
 
