# Supplementary material for: Detecting overlapping coding sequences in virus genomes
Source: BMC Bioinformatics. 2006 Feb 16;7:75. doi: 10.1186/1471-2105-7-75 (PMC1395342; doi:10.1186/1471-2105-7-75)
Supplement: Additional File 1 — Archive of the source code. The file sup1.TGZ is an archive of the source code for the current version of MLOGD. Unpack it with tar xvfz supl.TGZ; then see the README file in the MLOGD directory. [file 1471-2105-7-75-S1.TGZ › MLOGD/FORM/threshold2.html]

 
MLOGD: Notes


**Note on redrawing the
'Nucleotide-by-nucleotide' plots:**  
  
In the current 'Nucleotide-by-nucleotide' and zoomed-in
'Nucleotide-by-nucleotide' plots, the running mean of the log
likelihood scores summed over the input phylogenetic tree (i.e. the
input pairs file) skips regions that are gapped (or have ambiguous nt
codes) in any of the sequences in the pairs file. On the 'Redraw
plots' page, you will also have the option to extend this track into
gapped regions, provided the summed divergence of the contributing
sequence pairs in the region is greater than some user-defined
threshold value (details).  
  
 
