# Supplementary material for: Detecting overlapping coding sequences in virus genomes
Source: BMC Bioinformatics. 2006 Feb 16;7:75. doi: 10.1186/1471-2105-7-75 (PMC1395342; doi:10.1186/1471-2105-7-75)
Supplement: Additional File 1 — Archive of the source code. The file sup1.TGZ is an archive of the source code for the current version of MLOGD. Unpack it with tar xvfz supl.TGZ; then see the README file in the MLOGD directory. [file 1471-2105-7-75-S1.TGZ › MLOGD/FORM/error.html]

 
MLOGD: Notes


---

**Typical error messages include:**  

---

Warning message:  
zero-length arrow is of indeterminate angle and so skipped  
null device  
 1  
  
These come from the statistics package R, not from
MLOGD. They may be safely ignored.

---

Device png256 is not available  
  
This comes from the statistics package R, not from
MLOGD. It means that your version of R doesn't
recognize png256 and, as a result, fails to produce the png versions
of the plots. However, you should still get the eps and pdf versions
of the plots.

---

Aborting: sequence seq.003.fasta, unknown nucleotide 'z', at
2345.  
  
The programmes only accept ACGT and standard ambiguous nucleotide codes
MRWVHDBSYKNX, upper or lower case, plus '-' and '.' for gaps. seq.003.fasta
is the 3rd sequence in the input alignment.

---

Fitting number of mutations (87) outside that allowed by given
t range. Sequences 2 and 4.  
Iteration on t failed to converge in 20 iterations. Sequences
4 and 5.  
  
These error messages refer to fitting the *t* (pairwise sequence
divergence) parameter. Generally the results should be fine, even if
you get error messages. However you could check whether you have a
bad alignment - perhaps one of your sequences is too divergent from
the others.

---

Warning: gap within null model coding region not a multiple of
three nt; sequence 3, sequence coords 1280, alignment coords
1296.  
Warning: gap within alternative model coding region not a multiple
of three nt; sequence 3, sequence coords 1280, alignment coords
1296.  
  
There may be local problems with the various statistics and stop/start
codon annotation, as codons may be picked from the wrong read-frame.
Provided there aren't too many of these errors, the results should
still be basically OK. Otherwise you may need to redo your alignment,
insuring that, as far as possible, gaps within coding sequences occur
in groups of three. You can use the alignment site to do this.

---

Warning: orfs.1 has overlapping ORFs. Only the first will be used in
overlap regions.  
  
If either the input 'Known CDS(s)' (null model, orfs.1) or
input 'Query CDS(s)' (alternate model, orfs.2) contains
overlapping ORFs then, in the overlap regions, only the first ORF will
be used in the respective model.

---

Warning: sequence seq.001.fasta, ambiguous nt 'm', at 5.  
  
The ambiguous nucleotide, and any codons containing the ambiguous
nucleotide, will be omitted from the calculation of statistics, but
there should be no other ill effects.

---

Warning: failed to reach requested number of mutations; sequence:
99.  
  
This warning may occur in the Monte Carlo simulation programme for
very divergent sequences. Mutations are applied until the required
number of mutations are accepted, but there is a time-out switch in
case obtaining the required number of accepted mutations takes too
long.

---

**More serious error messages include:**  

---

Aborting: ORF 3 in orfs.1, endpoints outside sequence
range.  
  
Check the CDS annotation. Remember that these should be in the
ungapped reference sequence coordinates, not alignment coordinates.
orfs.1 refers to the 'Known CDS(s)' input while
orfs.2 refers to the 'Query CDS(s)' input.

---

Warning: number of nucleotides in ORF 1 in orfs.2 not a multiple
of 3.  
  
The programme will continue to work, but you should check your CDS
annotation. Note that for frameshifts, disjoint CDSs and circular
genomes, you should use the NCBI GenBank
'join(2307..3212,1..1623)' format in the CDS annotation.

---

Aborting: gapped sequences of differing length.  
  
Check your input alignment. All sequences, when gap characters are
included, must be of the same length.

---

Detected an ambiguous nt code in the reference sequence.
Can't run Monte Carlo simulations in this situation.  
  
The Monte Carlo simulation can not run if the initial sequence (the
reference sequence) contains ambiguous nucleotide codes. Choose a
different reference sequence, or else fudge the results by picking a
random nucleotide (A, C, G or T) for each ambiguous nucleotide in the
reference sequence.

---

 
