# Supplementary material for: Detecting overlapping coding sequences in virus genomes
Source: BMC Bioinformatics. 2006 Feb 16;7:75. doi: 10.1186/1471-2105-7-75 (PMC1395342; doi:10.1186/1471-2105-7-75)
Supplement: Additional File 1 — Archive of the source code. The file sup1.TGZ is an archive of the source code for the current version of MLOGD. Unpack it with tar xvfz supl.TGZ; then see the README file in the MLOGD directory. [file 1471-2105-7-75-S1.TGZ › MLOGD/FORM/frames.html]

 
MLOGD: Notes


**Notes on read-frame notation:**  
  
While the meaning of the forward read-direction frames +1 and +2
(relative to a reference +0 frame sequence) is clear, the meaning of
the -1 and -2 frames is less clear. In fact -1 and -2 are sometimes
used to describe forward read-direction frames - e.g. for
frameshifting, where a -1 frameshift corresponds to the +2 frame and a
-2 frameshift corresponds to the +1 frame. There is no general
standard for naming the reverse read-direction frames. Our convention
is illustrated in the figure below. In our system, the -1 frame
corresponds to the same frame as +1 but read in the reverse direction,
the -2 frame corresponds to the +2 frame but read in the reverse
direction, and the -0 or -3 frame corresponds to the +0 frame but read
in the reverse direction. So the number represents the register,
while the sign represents the read direction.  
  


  
  
The following table compares some different naming conventions for the
different possible read-frames of a second ORF relative to an initial
+0 frame ORF:  
  


|  |  |  |  |  |  |  |
| --- | --- | --- | --- | --- | --- | --- |
| Firth & Brown (2005) | 0 | +1 | +2 | -1 | -2 | -0/-3 |
| Smith & Waterman (1980) | 0 | 1 | 2 | 4 | 5 | 3 |
| Krakauer (2000) | - | +1 | +2 | -2 | -1 | 0 |
| Rogozin *et al.* (2002) | - | - | - | C2 | C1 | C3 |

  
  
If ORF A is in frame -0/-3 relative to ORF B, then it is easy to see
that ORF B is in frame -0/-3 relative to ORF A. This reciprocity is
true also for the -2 and -1 frames. However if ORF A is in frame +1
relative to ORF B, then ORF B is in frame +2 relative to ORF A and
*vice versa*. These relationships are summarized in the
following table. If ORF2 is in frame A relative to ORF1, then ORF1 is
in frame B relative to ORF2:  
  


| A | B |
| --- | --- |
| +1 | +2 |
| +2 | +1 |
| 0 | 0 |
| -0/-3 | -0/-3 |
| -1 | -1 |
| -2 | -2 |

  
  
**References:**

- Firth A. E., Brown C. M., 2005, Detecting overlapping coding
  sequences with pairwise alignments, *Bioinformatics*, **21**,
  282-92.- Krakauer D. C., 2000, Stability and evolution of overlapping
    genes, *Evolution*, **54**, 731-739.- Rogozin I. B. *et al.*, 2002, Purifying and directional
      selection in overlapping prokaryotic genes, *Trends Genet.*,
      **18**, 228-232.- Smith T. F., Waterman M. S., 1980, Protein constraints induced
        by multiframe encoding, *Math. Biosci.*, **49**, 17-26.
 
