# Supplementary material for: Detecting overlapping coding sequences in virus genomes
Source: BMC Bioinformatics. 2006 Feb 16;7:75. doi: 10.1186/1471-2105-7-75 (PMC1395342; doi:10.1186/1471-2105-7-75)
Supplement: Additional File 1 — Archive of the source code. The file sup1.TGZ is an archive of the source code for the current version of MLOGD. Unpack it with tar xvfz supl.TGZ; then see the README file in the MLOGD directory. [file 1471-2105-7-75-S1.TGZ › MLOGD/FORM/changes.html]

 
MLOGD: Notes


**Minor changes to algorithms since Firth &
Brown, 2005, *Bioinformatics*, **21**, 282-92:**  
  

- The divergence parameter *t* is now fitted to make the
  expected number of mutations under the model equal to the observed
  number of mutations. Previously, *t* was fitted to maximize
  the probability of the observed mutations under the model.  
    
  - A non-coding model has been added to the single and double
    coding models so it is now easy to analyse query CDSs that overlap
    other CDSs or non-coding regions or both.  
      
    - Codons that cross gapped regions in the alignment are no longer
      skipped.  
        
      - The required format of the input sequences and CDS annotation
        has been simplified.
 
