# Supplementary material for: Detecting overlapping coding sequences in virus genomes
Source: BMC Bioinformatics. 2006 Feb 16;7:75. doi: 10.1186/1471-2105-7-75 (PMC1395342; doi:10.1186/1471-2105-7-75)
Supplement: Additional File 1 — Archive of the source code. The file sup1.TGZ is an archive of the source code for the current version of MLOGD. Unpack it with tar xvfz supl.TGZ; then see the README file in the MLOGD directory. [file 1471-2105-7-75-S1.TGZ › MLOGD/FORM/falsepos.html]

 
MLOGD: Notes


**Note on read-frame combinations that can lead
to false positive signals:**  
  
The following read-frame combinations can sometimes result in false
positive signals (read-frame notation):

- A query ORF overlapping a known CDS, with the query ORF in the
  -2 frame relative to the +0 frame known CDS, is liable to produce a
  positive signal even when the query ORF is non-coding. This is
  because, in the -2 frame, N3 (the 3rd nucleotide position in codons)
  in the query ORF aligns with N3 in the known CDS. The pattern of
  mutations for two CDSs overlapping in these frames is 'highly
  variable N3 and constrained N1 and N2' (the 1st and 2nd nucleotide
  position in codons). This is similar to the pattern of mutations
  for a single non-overlapping CDS.  
    
  In the six-frame plots, the read-frame combinations to be wary of are:
    
    


  | Known CDS (coding) | Query CDS (false +ve) |
  | --- | --- |
  | +0 | -2 |
  | +1 | -0/-3 |
  | +2 | -1 |
  | -0/-3 | +1 |
  | -1 | +2 |
  | -2 | +0 |

    
  The presense or absense of stop codons can be a useful additional
  clue as to whether such a region of positive signal is or isn't
  coding.  
    
  - When there are two overlapping known CDSs, in the overlap region
    only the first CDS in the input 'Known CDSs' list will be
    incorporated into the null model. In the six-frame plots, the other
    CDS should show up as a positive signal. In addition, there are
    certain read-frame combinations of the two known CDSs that may lead
    to a false positive signal in a third frame. These are as
    follows (for Known CDS 1 in the forward read-direction):  
      


    | Known CDS 1 (coding) | Known CDS 2 (coding) | Query CDS (false +ve) |
    | --- | --- | --- |
    | +0 | -0/-3 | +1 |
    | +0 | +1 | -0/-3 |
    | +0 | +2 | -1 |
    | +0 | -1 | +2 |
    | +1 | -1 | +2 |
    | +1 | +2 | -1 |
    | +1 | +0 | -2 |
    | +1 | -2 | +0 |
    | +2 | -2 | +0 |
    | +2 | +0 | -2 |
    | +2 | +1 | -0/-3 |
    | +2 | -0/-3 | +1 |

      
    The presense or absense of stop codons can be a useful additional
    clue as to whether such a region of positive signal is coding.
    Aside from that, while these particular read-frame combinations may
    give a false positive signal when the Query CDS is tested against
    Known CDS 1, they are all expected to give a negative signal when
    the Query CDS is tested against Known CDS 2, and thus may be
    selected out in this way.
 
