# Supplementary material for: Detecting overlapping coding sequences in virus genomes
Source: BMC Bioinformatics. 2006 Feb 16;7:75. doi: 10.1186/1471-2105-7-75 (PMC1395342; doi:10.1186/1471-2105-7-75)
Supplement: Additional File 1 — Archive of the source code. The file sup1.TGZ is an archive of the source code for the current version of MLOGD. Unpack it with tar xvfz supl.TGZ; then see the README file in the MLOGD directory. [file 1471-2105-7-75-S1.TGZ › MLOGD/FORM/nullmodneg.html]

 
MLOGD: Notes


**Note on null and alternate model:**  
  
If one of the Query CDSs overlaps *in the same read-frame* one of
the input 'Known CDS(s)', then don't be surprised if the query CDS
gives a negative signal. Such CDSs have already been taken into
account as part of the null model. The negative signal supports the
null model (i.e. that only the 'Known CDS(s)' are coding) rather than
the alternate model (effectively that the Query CDS is coding
'twice').
 
