# Supplementary material for: Detecting overlapping coding sequences in virus genomes
Source: BMC Bioinformatics. 2006 Feb 16;7:75. doi: 10.1186/1471-2105-7-75 (PMC1395342; doi:10.1186/1471-2105-7-75)
Supplement: Additional File 1 — Archive of the source code. The file sup1.TGZ is an archive of the source code for the current version of MLOGD. Unpack it with tar xvfz supl.TGZ; then see the README file in the MLOGD directory. [file 1471-2105-7-75-S1.TGZ › MLOGD/FORM/guidedtour.html]

 
MLOGD: Notes


**Example full output for the different
running modes:**  

- 'Test input query CDSs'
  (Hepatitis B Virus - P, C, X genes taken as the Known CDSs, overlapping S
  gene taken as the Query CDS.)- 'Find and test all
    non-annotated ORFs' (Enterovirus with single long poly-protein
    CDS annotated.)- 'Six-frame sliding window
      plots' (Luteovirus with no Known CDSs annotated.)

  
  
**Guided tour:**  

1. Suppose you are interested in Hepatitis B Virus NCBI GenBank accession
   number AB014366. The first step is to obtain a set of related
   sequences. You could do this via the NCBI blast site.
   Alternatively, for viruses, you could use the NCBI viral 
   genomes or taxonomy
   databases.  
     
   - Align the sequences and obtain a phylogenetic tree pairs file
     here. Alternatively, you
     could use e.g. PHYLIP to calculate a phylogenetic tree and convert
     it a pairs file here.   
       
     - Annotate the known CDSs in the reference sequence. E.g. for
       AB014366 you can use the NCBI GenBank annotation
       (the CDS lines). Be aware that the GenBank annotation is sometimes
       incomplete.  
         
       - Now you are ready to go to the MLOGD base page here.  
           
         - If you have a specific query CDS (e.g. a long ORF that has not
           previously been annotated as a CDS, but that you suspect may be a
           CDS) then use the 'Test input query CDSs' option. Input the query
           CDS location and click on 'Calculate'. The results page gives:
             
             
           - Null versus alternate model likelihood ratio statistics,
             where the null model is that the query ORF is not coding while the
             alternate model is that the query ORF is coding (both the null and
             alternate models include the annotated CDSs).  
               
             - A plot of the likelihood ratio statistics (example plot). You may follow a link
               here to generate simulated sequences under the same null and
               alternate models. The simulations are used to put error bars on
               the likelihood ratio statistics, and to interpret the
               synonymous/nonsynonymous and N1/N2/N3 statistics (example plot).  
                 
               - A nucleotide-by-nucleotide plot of the likelihood ratio
                 statistic for each reference - non-reference sequence pair and
                 summed over the phylogenetic tree (example
                 plot). Gaps and stop and start codons for each sequence are also
                 annotated on the plot. You may follow a link here to zoom in on
                 the plot, add grid lines, or adjust the running-mean window size.
                 There is also a zoomed in version of this plot (example plot).- If you don't have a specific query CDS, you may either select
             the 'Find and test all non-annotated ORFs' option, or select the
             'Six-frame sliding window plots' option.  
               
             - The 'Find and test all non-annotated ORFs' option will find all
               non-annotated ORFs and produce the same statistics and plots as the
               'Test input query CDSs' option for each of these ORFs.  
                 
               - The 'Six-frame sliding window plots' option will calculate the
                 MLOGD statistic in a window sliding along the alignment in all six reading
                 frames (example plot). Extended regions
                 of positive signal may indicate potential new CDSs, especially where
                 there is an absense of stop codons. Once you have located any
                 potentially interesting regions, you can then go back to the 'Test
                 input query CDSs' option to investigate these regions in more
                 detail.
 
