# Supplementary material for: Detecting overlapping coding sequences in virus genomes
Source: BMC Bioinformatics. 2006 Feb 16;7:75. doi: 10.1186/1471-2105-7-75 (PMC1395342; doi:10.1186/1471-2105-7-75)
Supplement: Additional File 1 — Archive of the source code. The file sup1.TGZ is an archive of the source code for the current version of MLOGD. Unpack it with tar xvfz supl.TGZ; then see the README file in the MLOGD directory. [file 1471-2105-7-75-S1.TGZ › MLOGD/FORM/probint.html]

 
MLOGD: Notes


**Note on interpretation of the log likelihood
ratio:**  
  
The probabiliy ratio Prob(alt)/Prob(null) quoted on the 'Test input
query CDSs' results page is calculated as  
  
Prob(alt)/Prob(null) ~ exp(ln(LR))  
  
where ln(LR) is the log likelihood ratio summed over all
contributing nucleotides in each sequence pair, and summed over the
sequence pairs tracing round the phylogenetic tree
(as described here).  
  
Converting the likelihood ratio to a probability like this involves
the following assumptions:

- The null and alternate model have equal Bayesian prior probabilities.- The individual likelihood ratios (i.e. for a given nucleotide
    position in a given aligned sequence pair) are all independent. The
    dependence on phylogeny is dealt with as described here. However the likelihood ratios for
    different nucleotide positions are unlikely to be independent:
    adjacent nucleotide positions can be linked by being in the same
    codon; distal nucleotide positions can be linked by protein or RNA
    secondary structure interactions; and nucleotides at the same
    position in different sequence pairs can be linked by similar
    selective pressure on an encoded protein amino acid position. (The
    effect of non-independence is just to exaggerate the log likelihood
    ratio, rather than change its sign.)- The mutation model is accurate.

Since these assumptions are unlikely to be met, the quoted
'probability ratios' should not be taken too seriously.
 
