# Supplementary material for: Detecting overlapping coding sequences in virus genomes
Source: BMC Bioinformatics. 2006 Feb 16;7:75. doi: 10.1186/1471-2105-7-75 (PMC1395342; doi:10.1186/1471-2105-7-75)
Supplement: Additional File 1 — Archive of the source code. The file sup1.TGZ is an archive of the source code for the current version of MLOGD. Unpack it with tar xvfz supl.TGZ; then see the README file in the MLOGD directory. [file 1471-2105-7-75-S1.TGZ › MLOGD/FORM/plot.mletrack.html]

  MLOGD: Notes   
**Notes on the zoomed in 'Nucleotide-by-nucleotide'
plot:**  
  
This is a plot of the likelihood ratio, summed over the input
phylogenetic tree (details), at single nucleotide
resolution. The five panels show the following information:

1. This panel displays the raw likelihood ratio scores, summed over
   the phylogenetic tree, at each column position in the input
   alignment.  
     
   - This panel displays the running mean (i.e. sliding window mean) of
     the likelihood ratio scores. The sliding window width is annotated
     on the plot, and can be changed via the 'Redraw plot' link.  
       
     - This panel shows the input Query, or alternate model, CDS(s) (red
       bars), the input Known, or null model, CDS(s) (blue bars), and the
       reference sequence nucleotide sequence.  
         
       - This panel shows the phylogenetic sum of sequence divergences
         (mean number of mutations per nucleotide) for the sequence pairs
         that contribute to the likelihood ratio sum at each position in the
         alignment. In any particular column, some sequences may be omitted
         from the likelihood ratio calculations due to gaps or stop to
         non-stop transitions. Statistics in regions with lower summed
         divergence (i.e. partially gapped regions) have a lower
         signal-to-noise ratio.  
           
         - This panel shows the alignment and reference sequence
           coordinates of each position.

**Notes:**

- For panel 2, any columns with gaps (or ambiguous nt codes) in
  any sequence in the input list of sequence pairs are omitted. Such
  columns are omitted before taking the running mean. Thus where gaps
  occur a, for example, 21 nt window includes a total of 21 columns
  taken from either side of the gap but none from within the gap. On
  the 'Redraw plots' page, you may choose to extend the plot into
  partially gapped regions, provided the summed divergence of the
  contributing sequence pairs in the region is greater than some
  user-defined threshold value (details).
 
