# Supplementary material for: Detecting overlapping coding sequences in virus genomes
Source: BMC Bioinformatics. 2006 Feb 16;7:75. doi: 10.1186/1471-2105-7-75 (PMC1395342; doi:10.1186/1471-2105-7-75)
Supplement: Additional File 1 — Archive of the source code. The file sup1.TGZ is an archive of the source code for the current version of MLOGD. Unpack it with tar xvfz supl.TGZ; then see the README file in the MLOGD directory. [file 1471-2105-7-75-S1.TGZ › MLOGD/FORM/virus.html]

 
MLOGD: Notes


**Files and plots in the Virus
Database:**  
  
The columns of the table contain links to the following files:

1. **name:** Species name.  
     
   - **sequences:** List of the strains used. First strain is
     taken as the reference sequence.  
       
     - **alignment:** Sequence alignment.  
         
       - **pairs:** List of sequence pairs tracing round the perimeter
         of one possible phylogenetic tree (details).  
           
         - **known CDSs:** Positions of known CDSs.  
             
           - **six-frame (null model = non-coding):** The MLOGD six-frame
             plots (40 codon window, 10 codon step size), taking the null model
             to be that the whole genome is non-coding. In general, the known
             CDSs should show up as regions of positive signal. In overlapping
             CDS regions (e.g. in Hepatitis B Virus) there can be some confusion
             in the signal, depending on the respective read-frames.  
               
             - **six-frame (null model = known CDSs):** The MLOGD six-frame
               plots (40 codon window, 10 codon step size), taking the known CDSs
               as the null model. In these plots, the known CDSs won't necessarily
               have a positive signal since they have already been taken into
               account in the null model. Extended regions of positive signal may
               indicate potential new CDSs, especially where there is an absense of
               stop codons. Note that if there are overlapping CDSs in the list of
               known CDSs, then only the first CDS will be used in the null model
               within the region of overlap; the second CDS should then show up as
               a positive signal (e.g. for the first Hepatitis B Virus alignment,
               the P gene (487..3018) is in the null model, so has a low or
               negative signal in the six-frame plot, while the overlapping S gene
               (1028..2230) is not in the null model, so has a positive signal in
               the six-frame plot).  
                 
               - **annotated CDSs (null model = non-coding):** This contains
                 links to plots and statistics for each annotated known CDS. For
                 each CDS, the null model is that the whole genome is non-coding,
                 while the alternate model is that the annotated CDS is coding. For
                 each CDS, there are tables of the MLOGD and other statistics, plots
                 of the MLOGD statistic summed over the sequences and at
                 single-nucleotide resolution, and Monte Carlo simulation statistics
                 and plots.  
                   
                 - **annotated CDSs (null model = other CDSs):** This contains
                   links to plots and statistics for each annotated known CDS. For
                   each CDS, the null model is that the CDS is non-coding but all the
                   other known CDSs are coding, while the alternate model is that all
                   the known CDS are coding. (For genomes with a single CDS, this is
                   the same as 'annotated CDSs (null model = non-coding)'.) For each
                   CDS, there are tables of the MLOGD and other statistics, plots of
                   the MLOGD statistic summed over the sequences and at
                   single-nucleotide resolution, and Monte Carlo simulation statistics
                   and plots.  
                     
                   - **all non-annotated ORFs:** This contains links to tables of
                     the MLOGD and other statistics, and plots of the MLOGD statistic
                     summed over the sequences and at single-nucleotide resolution, for
                     each non-annotated start-stop ORF >= 40 codons in the reference
                     sequence. For each ORF, the null model is that only the known CDSs
                     are coding, while the alternate model is that both the known CDSs
                     and the query ORF are coding.
 
