# Supplementary material for: Detecting overlapping coding sequences in virus genomes
Source: BMC Bioinformatics. 2006 Feb 16;7:75. doi: 10.1186/1471-2105-7-75 (PMC1395342; doi:10.1186/1471-2105-7-75)
Supplement: Additional File 1 — Archive of the source code. The file sup1.TGZ is an archive of the source code for the current version of MLOGD. Unpack it with tar xvfz supl.TGZ; then see the README file in the MLOGD directory. [file 1471-2105-7-75-S1.TGZ › MLOGD/FORM/plot.mcsims.html]

 
MLOGD: Notes


**Notes on the 'Monte Carlo simulations' plot:**  
  
The rows of the plot show the real data and the simulations for the
following statistics:

1. MLOGD log likelihood ratio per nucleotide. (I.e. the same as the
   'Likelihood ratio plot' on the initial MLOGD results page, or column
   6 of the 'Statistics summary' table on the initial MLOGD results
   page.)  
     
   - Fraction of the aligned codons, within the null model CDSs, that
     are different between the two sequences but code for identical amino
     acids. (I.e. synonymous codon mutation fraction, or column 8 of the
     'Statistics summary' table on the initial MLOGD results page.)  
       
     - Fraction of the aligned codons, within the null model CDSs, that
       are different between the two sequences and code for different amino
       acids. (I.e. nonsynonymous codon mutation fraction, or column 9 of
       the 'Statistics summary' table on the initial MLOGD results page.)  
         
       - Fraction of the 1st codon position nucleotides (e.g. the C in
         CAG *gln*), within the null model CDSs, that are different between the
         two sequences. (I.e. 1st codon position mutation fraction, or column
         10 of the 'Statistics summary' table on the initial MLOGD results
         page.)  
           
         - Fraction of the 2nd codon position nucleotides (e.g. the A in
           CAG *gln*), within the null model CDSs, that are different between the
           two sequences. (I.e. 2nd codon position mutation fraction, or column
           11 of the 'Statistics summary' table on the initial MLOGD results
           page.)  
             
           - Fraction of the 3rd codon position nucleotides (e.g. the G in
             CAG *gln*), within the null model CDSs, that are different between the
             two sequences. (I.e. 3rd codon position mutation fraction, or column
             12 of the 'Statistics summary' table on the initial MLOGD results
             page.)

The horizontal axis on all plots shows sequence divergence (mean
number of mutations per column). For the 'sum over tree' column, this
is the summed divergence over the input list of sequence
pairs.  
  
The first two columns plot the statistics for reference -
non-reference sequence pairs (red circles). These are compared with
the distribution of scores from the simulated sequence pairs using the
null model annotation (first column, green dots and black error bars),
and using the alternate model CDS annotation (second column, purple
dots and black error bars). The green and purple dots show the
general distribution of scores as a function of sequence divergence
(GD simulations), while the error bars are based on simulations at the
same pairwise divergence from the reference sequence as for the
corresponding red circle (EB simulations).  
  
Note that the red circles may not coincide with either model. This
just indicates that the assumed mutation model doesn't precisely
reflect the actual mutation probabilities. For example, there is no
synonymous:nonsynonymous ratio fitting parameter in the model (there
are good reasons for not including this). If a CDS is particularly
highly conserved (due to protein constraints), then it may get higher
MLOGD scores than the model predictions. Conversely, if a CDS is
particularly variable, it may get lower MLOGD scores than the model
predictions. However, the purpose of MLOGD is not to
as-accurately-as-possible model sequence evolution, but just to be
able to distinguish a coding sequence from a non-coding sequence (or a
double-coding sequence from a single-coding sequence). For this
purpose, the MLOGD mutation model is quite adequate.  
  
A given observation (one of the red circles on the plot) is taken as
support for either the null or the alternate model, depending on which
of the two simulated distributions it is closest to, in units of the
standard deviation of the respective simulated distributions. By
assuming that the statistics for the simulated data sets are normally
distributed, the corresponding likelihood ratio may be calculated (details). These likelihood ratios, derived
for each of the six statistics (MLOGD and the N1, N2, N3, synonymous
and nonsynonymous mutation fractions), are summarized in the table at
the bottom of the results page.  
  
The third column in the plot shows the 'summed over phylogenetic tree'
(i.e. summed over the input list of sequence pairs) scores (details). Error bars are calculated by taking a
similar sum over pairs for simulated sequences. Once again, one of
the blue circles on the plot is taken as support for either the null
or the alternate model, depending on which of the two simulated
distributions it is closest to, in units of the standard deviation of
the respective simulated distributions. The corresponding likelihood
ratios are summarized at the bottom of the table on the results
page.  
  
  
**Notes:**

- The synonymous, nonsynonymous and 1st/2nd/3rd codon position
  data are calculated for the null model CDS regions only. If there
  are no CDSs annotated in the null model then the data in rows 2-6 of
  the plot will be identically zero.
 
