# Supplementary material for: Detecting overlapping coding sequences in virus genomes
Source: BMC Bioinformatics. 2006 Feb 16;7:75. doi: 10.1186/1471-2105-7-75 (PMC1395342; doi:10.1186/1471-2105-7-75)
Supplement: Additional File 1 — Archive of the source code. The file sup1.TGZ is an archive of the source code for the current version of MLOGD. Unpack it with tar xvfz supl.TGZ; then see the README file in the MLOGD directory. [file 1471-2105-7-75-S1.TGZ › MLOGD/SCRIPTS/mlenuc.plotdata.html]

 
MLOGD: Notes


**Raw plot data for 'Nucleotide-by-nucleotide'
plot:**  
  
  
Likelihood scores:  

1. Sequence 1 ID number. (Always equal to '1' - i.e. the reference
   sequence.)- Sequence 2 ID number. (Order is the same as the rows in the
     'Statistics summary' table, starting with '2'.)- Alignment coordinate (first nucleotide in the alignment is '1'.)- Log likelihood score for the null model.- Log likelihood score for the alternate model.

Note that entries for alignment coordinates corresponding to
nucleotides which are gapped (or ambiguous nt codes) in either
sequence 1 or sequence 2, or which are members of codons involved in
stop to non-stop codon transitions, in either the null or alternate
model CDSs, are omitted.  
  
  
Likelihood scores summed over
phylogenetic tree:  

1. Alignment coordinate.- Number of contributing sequence pairs (e.g. not gapped at this
     position).- Sum of pairwise sequence divergences (mean number of mutations
       per nucleotide) for the contributing sequence pairs, divided by four.- Sum of log likelihood scores for the null model for the
         contributing sequence pairs, divided by four.- Sum of log likelihood scores for the alternate model for the
           contributing sequence pairs, divided by four.- Reference sequence coordinate.- Reference sequence nucleotide.

Note that, outside the query region, columns 2-5 are zero. See this
note on summing scores over a
phylogenetic tree; in particular for why columns 3-5 are divided by
four.  
  
  
Stop codon positions:  

1. Model (0 = null model, 1 = alternate model).- Sequence ID.- Alignment coordinate.

  
Start codon positions:  

1. Model (0 = null model, 1 = alternate model).- Sequence ID.- Alignment coordinate.

  
Gap (and ambiguous nt) positions:  

1. Sequence ID.- Alignment coordinate.
 
