# Supplementary material for: Detecting overlapping coding sequences in virus genomes
Source: BMC Bioinformatics. 2006 Feb 16;7:75. doi: 10.1186/1471-2105-7-75 (PMC1395342; doi:10.1186/1471-2105-7-75)
Supplement: Additional File 1 — Archive of the source code. The file sup1.TGZ is an archive of the source code for the current version of MLOGD. Unpack it with tar xvfz supl.TGZ; then see the README file in the MLOGD directory. [file 1471-2105-7-75-S1.TGZ › MLOGD/SCRIPTS/sixframe.plotdata.html]

 
MLOGD: Notes


**Raw plot data for 'Six-frame' plot:**  
  
  
Likelihood ratios:  

1. Frame.- Alignment coordinate of window centre.- Likelihood ratio: 'Window is coding in addition to the input
       Known CDS(s)' versus 'Only the input Known CDS(s) are coding'.- Alignment coordinate of window start.- Alignment coordinate of window end.- Sum over sequence pairs of (number of nt used in window) x
             (pairwise sequence divergence), where here the sequence divergence
             of each pair is the mean number of mutations per nucleotide for
             the whole sequence (rather than just in the window), and the
             number of nt used is generally equal to the window length, but may
             be reduced for gaps, ambiguous nt codes or stop to non-stop
             transitions. This is basically a measure of how much data is
             available in each window to calculate the likelihood ratio statistic;
             lower values correspond to lower signal-to-noise.

  
Stop codon positions:  

1. Frame.- Sequence ID.- Alignment coordinate.

  
Start codon positions:  

1. Frame.- Sequence ID.- Alignment coordinate.

  
Gap (and ambiguous nt) positions:  

1. Sequence ID.- Alignment coordinate.

  
Summed sequence divergences:  

1. Alignment coordinate.- Divergence of contributing sequence pairs (i.e. not gapped etc), summed
     over phylogenetic tree.
 
