# Supplementary material for: Detecting overlapping coding sequences in virus genomes
Source: BMC Bioinformatics. 2006 Feb 16;7:75. doi: 10.1186/1471-2105-7-75 (PMC1395342; doi:10.1186/1471-2105-7-75)
Supplement: Additional File 1 — Archive of the source code. The file sup1.TGZ is an archive of the source code for the current version of MLOGD. Unpack it with tar xvfz supl.TGZ; then see the README file in the MLOGD directory. [file 1471-2105-7-75-S1.TGZ › MLOGD/SCRIPTS/mcsim.plotdata.html]

 
MLOGD: Notes


**Raw plot data for the 'Monte Carlo simulations'
plot:**  
  
Original data for reference -
non-reference sequence pairs.  
  
Error bar data.  
  
General distribution of statistics for null
model simulations.  
  
General distribution of statistics for alternate model simulations.  
  
 
